# Age‐Trajectory of Mother–Infant Relationships in Wild Assamese Macaques

**DOI:** 10.1002/ajp.70110

**Published:** 2026-01-20

**Authors:** Ana Lucia Arbaiza‐Bayona, Roger Mundry, Suchinda Malaivijitnond, Suthirote Meesawat, Oliver Schülke, Julia Ostner

**Affiliations:** ^1^ Department of Behavioral Ecology, Johann‐Friedrich‐Blumenbach Institute for Zoology and Anthropology Georg‐August‐Universität Göttingen Göttingen Germany; ^2^ Research Group Primate Social Evolution, German Primate Center, Leibniz Institute for Primate Research Göttingen Germany; ^3^ Leibniz ScienceCampus Primate Cognition, German Primate Center, Leibniz Institute for Primate Research Göttingen Germany; ^4^ Department for Primate Cognition, Johann‐Friedrich‐Blumenbach Institute Georg‐August‐Universität Göttingen Göttingen Germany; ^5^ Cognitive Ethology Laboratory, German Primate Center, Leibniz Institute for Primate Research Göttingen Germany; ^6^ National Primate Research Center of Thailand Chulalongkorn University Saraburi Thailand; ^7^ Department of Biology, Faculty of Science Chulalongkorn University Bangkok Thailand

**Keywords:** ecological constraints, infant independence, maternal investment, mother–infant spatial relationship, seasonal reproduction

## Abstract

Maternal care is ubiquitous in mammals, yet its degree and duration vary across taxa. In primates, mothers provide extended care for young and follow similar developmental transitions in the mother–infant relationship, yet at different paces of change. Since ecological pressures shape life‐history traits including female reproductive rate and timing of infant independence, research is needed on mother–infant relationships in wild populations exposed to energetic constraints and predation risk. Assamese macaques (*Macaca assamensis*) of the study population are seasonal breeders living in an unpredictable environment, where fluctuating food availability imposes energetic challenges on mothers and infants. We quantitatively describe how maternal care and offspring independence develop throughout infancy. Using continuous focal observations on 59 infants, we model the nonlinear age‐trajectories of mother–infant proximity and transitions from dependent to independent feeding and locomotion, and estimated sex differences in these trajectories. Newborns were fully dependent on their mothers for feeding and transport, with mothers maintaining close proximity. A transitional phase emerged between 1 and 3 months of age, marked by reduced maternal proximity and increasing infant independence. During the second half of infancy, infants achieved near‐complete locomotor and feeding independence, while residual proximity and body contact persisted. No sex differences were detected in the mother–infant relationship trajectory. Collectively, the timing of maternal investment aligns with the breeding strategy of this seasonal species, with females balancing investment in current and future reproduction. This study establishes a baseline for examining how ecological variability affects the timing and pace of mother–infant behavioral transitions.

## Summary

- Maternal care was initially intense, dropped sharply between 1 and 3 months of infant age, and stabilized at a lower level during the second half of infancy.
- Infant sex did not influence the mother–infant relationship.
- Early onset of infant independence was associated with female reproductive strategies that reallocate investment from current offspring toward future reproduction.

## Graphical abstract

Maternal care in wild Assamese macaques declined sharply between 1 and 3 months of infant age as infant independence increased, and stabilized from around 6 months, with no sex differences in the trajectory. This early shift aligns with the species' seasonal breeding strategy, with females balancing investment in current and future offspring.

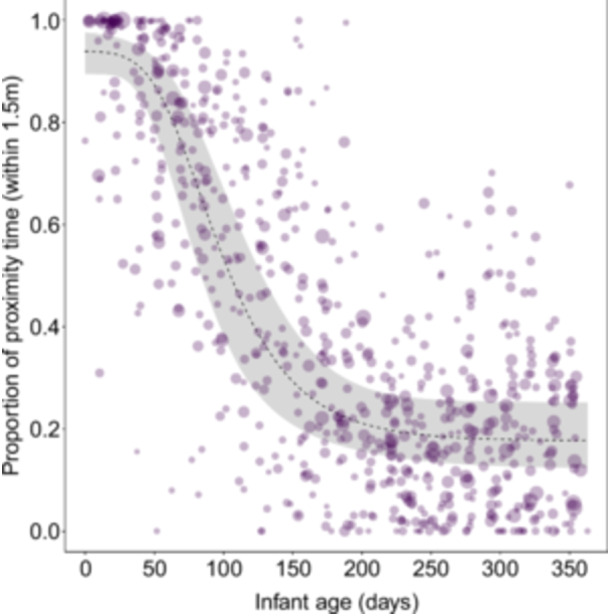

## 1 Introduction

Parental care is widespread in vertebrates but varies considerably among taxa in terms of who provides it, as well as how and for how long it is given (Gubernick and Klopfer 1981). Mammalian females invest heavily in gestation and lactation (Altmann and Samuels 1992; Gittleman and Thompson 1988), with varying degrees and forms of postnatal care. Primates, in particular, have a slow life history for their body size compared to other mammals and are characterized by prolonged maternal care (Charnov and Berrigan 1993; Zipple et al. 2024).

The interaction between an infant and its mother begins with the physiological connection established during gestation via the placenta and develops after birth into a close relationship that provides nutrition, protection, thermoregulation, and means of transportation (Fairbanks 2003; Nicolson 1991). Primate mothers may extend their care beyond weaning, sometimes continuing into later life stages (Crockford et al. 2020; van Noordwijk 2012), allowing ample opportunities for maternal influence on offspring development (Bardi and Huffman 2002; Maestripieri and Mateo 2009; Samuni et al. 2020). Moreover, the mother serves as a source of learning by offering safe opportunities for direct social learning (Estienne et al. 2019; Mikeliban et al. 2021; Sargeant and Mann 2009), and by acting as a secure base from which infants explore and learn about their ecological and social environment (Ainsworth 1979; Suomi 2005; Uomini et al. 2020). Primate mothers do not only provide intensive and extended care to their newborns, who rely on them for survival until they develop the necessary skills to become self‐sufficient (Zipple et al. 2020), but the quality and duration of their care can also shape future offspring survival and reproductive success (Stanton et al. 2020; Surbeck et al. 2010; Zipple et al. 2020), through multiple mechanisms (Marks and Lailvaux 2024).

The developmental trajectory of the mother–infant relationship is broadly shared among primates. When a primate is born, its mother maintains close contact and proximity, dedicating considerable time to lactation and carrying (Altmann and Samuels 1992; Amici et al. 2025; Castellano‐Navarro et al. 2023). This significant investment requires adjustments of the mother's activity budget to accommodate the corresponding increase in energy expenditure (Dittus and Baker 2024; Dunbar and Dunbar 1988; Touitou et al. 2021). As the infant matures and develops the necessary motor skills to move away from its mother (Berghänel et al. 2015), it begins to break contact and explore the ecological and social surroundings (Byrne and Suomi 1995; Castellano‐Navarro et al. 2023; Dittus and Baker 2024). However, to mitigate the risk of mortality from predation, intragroup aggression, or injury, the mother initially restrains her infant's attempts to move away and actively minimizes the distance sought by the offspring (R. A. Hinde and Spencer‐Booth 1968; Lycett et al. 1998; Maestripieri 1995; Roura‐Torres et al. 2025). Over time, the mother decreases the energetic investment in the infant, shifting energy allocation more toward her own maintenance and future reproduction (Dittus and Baker 2024; Shivani et al. 2025; Trivers 1974). The mother gradually increases the distance between herself and her infant, while refusing the infant's attempts to nurse or to be carried for transportation. In response, the infant aims to maximize its own growth and survival by displaying signs of distress to regain maternal care or by actively seeking contact and proximity with her (Maestripieri 1995). The conflict between maternal reproductive potential and infant survival develops across infancy and ultimately leads to the offspring's nutritional (i.e., weaning) and locomotor independence.

Although the sequence of changes of the mother–infant relationship described above is shared among different primate taxa, the pace of moving through these changes varies significantly across species and populations (Harvey and Clutton‐Brock 1985; Langer 2003; Young and Shapiro 2018). For instance, the timing of locomotory independence, defined as infants being carried < 20% of their daily traveling time, can vary from 35 days after birth in Senegal bushbabys (*Galago senegalensis*) to 1826 days after birth in chimpanzees (*Pan troglodytes*) and bonobos (*Pan paniscus*, Young and Shapiro 2018). Life‐history theory provides a framework to understand this variation (Stearns 1976), whereby smaller species and those with smaller relative brain size generally develop faster, and larger species and those with larger brain size develop more slowly (Barrickman et al. 2008; Lee et al. 1991; Sol 2009). Yet, life history traits are not only explained by allometric features but also by ecological factors such as the extrinsic mortality level (Clutton‐Brock and Janson 2012; Promislow and Harvey 1990) or the diet of a given population (Borries et al. 2011; Clutton‐Brock and Janson 2012; Janson and van Schaik 1993; Robbins et al. 2023). For example, differences in the developmental speed of lowland (*Gorilla gorilla*) and mountain gorillas (*Gorilla beringei*) have been explained by variation in feeding ecology (Robbins et al. 2023). The link between ecological conditions and life‐history traits highlights the importance of studying different populations exposed to diverse ecological pressures.

Life‐history traits such as the timing of independence and maternal investment reflect trade‐offs due to energetic conflicts within resource‐limited environments (Stearns 1976). For instance, Asian colobines and macaques in the wild exhibit longer gestation periods, longer interbirth intervals, and a later age at first birth compared to provisioned populations of the same species (Borries et al. 2011). Although the genus *Macaca* has been the focus of pioneering work on mother–infant relationships (R. A. Hinde and Davies 1972; Maestripieri 1995; Thierry 1985), behavioral data from macaque populations living in their natural habitats are still scarce. Here, we focus on a population of Assamese macaques (*M. assamensis*) to provide insights into how maternal care and offspring independence develop throughout infancy under natural conditions. To this end, we use methods from growth studies to quantitatively describe temporal changes in the mother–infant relationship.

Assamese macaques, like all macaques, live in multi‐male multi‐female groups with male dispersal and female philopatry (Thierry 2007). Reproduction is highly seasonal and females typically conceive their first offspring at around 5.5 years of age (Anzà et al. 2022; Fürtbauer et al. 2010). Food availability fluctuates considerably within and between years (Heesen et al. 2013), creating unpredictable ecological conditions for gestating and lactating females as well as developing infants (Smith et al. 2017). Females follow a relaxed income breeding strategy (Brockman and Schaik 2005) building up energy stores during late lactation (i.e., preconception) and having an increased probability of conception when being in better physical condition (Heesen et al. 2013; Touitou et al. 2021). Interbirth intervals are bimodally (1 or 2 years) distributed and the presence of a dependent (unweaned) offspring strongly decreases the probability to conceive in a given mating season (Shivani et al. 2025). If a mother conceives while nursing a dependent young, the survival probability of the dependent, nursed, infant is reduced, suggesting maternal energy allocation away from the present into the future offspring (Shivani et al. 2025). Collectively, these patterns indicate energetic trade‐offs between maternal and infant fitness in this population, imposed by an unpredictable and resource‐limited environment, which are likely reflected and possibly mediated by the development of the mother–infant relationship.

Our aim was to explore the age‐dependent trajectories of the mother–infant relationship in the study population of Assamese macaques in terms of (a) the spatial behavior of mothers and their infants toward each other and (b) the transition from dependent to independent feeding and locomotion. Given the altricial nature of primates, we expected high amounts of maternal care and minimal infant independence immediately after birth, when newborns are highly dependent on their mothers for feeding and transportation, and spend the majority of their time in body contact or close proximity to their mother. Based on previous descriptions of mother–infant relationships in wild macaques (e.g., Berman 1980; Dittus and Baker 2024; Lindburg 1971), we predicted a gradual decline in mother proximity and body contact seeking behaviors during the first half of infancy, accompanied by a steeper decrease in high‐investment maternal behaviors (i.e., carrying and nursing). At the same time, we expected a corresponding early and gradual increase in independent locomotion and feeding by the offspring. We further predicted that high‐investment maternal behaviors would largely cease by the end of infancy, possibly driven by an increasing rate of maternal rejection of infant contact attempts, consistent with the population's occasional 1‐year interbirth intervals. Nevertheless, we anticipated that some level of proximity and body contact would persist in later infancy, reflecting both the general spatial cohesion of group members and previous reports that offspring in this population continue to engage in nipple contact at the end of the first year (Berghänel et al. 2016). As we did not expect a linear age‐trajectory in maternal and infant behaviors, we used nonlinear modeling techniques to quantitatively describe the developmental transitions in the mother–infant relationship.

We further investigated whether the sex of an infant influenced the developmental trajectory of mother–infant relationships. In philopatric species, mothers tend to invest more in the resident sex due to the potential benefits of maintaining a social bond with individuals who will remain in the group. For example, in female‐philopatric rhesus macaques, mothers are more aggressive and less likely to groom or maintain body contact with male compared to female infants (Kulik et al. 2016), whereas in male‐philopatric black‐handed spider monkeys (*Ateles geoffroyi*, Symington 1987) and chimpanzees (Bădescu et al. 2022), mothers tend to invest more in male compared to female offspring. Given that high maternal investment during early infancy is necessary to ensure infant survival, we expected sex differences in maternal care and offspring independence to become more pronounced with increasing infant age. Consequently, based on the dispersal regime of macaques, we expected mothers of male infants to decrease carrying, nursing, and contact and proximity‐seeking behaviors earlier and/or faster than mothers of female infants. In conjunction, we expected that male infants exhibit an earlier increase in body contact and proximity‐seeking behaviors as well as their feeding and locomotion independence, compared to female infants. Finally, we expected that by the end of infancy, male infants would maintain lower spatial proximity to their mothers compared to female infants, while still showing some proximity due to occasional nursing events and continued infant proximity‐seeking behavior.

## 2 Methods

### 2.1 Ethics Statement

All protocols and procedures implemented in this study are in compliance with all legal regulations for research in Thailand and Germany. Data collection was authorized by the Department of National Parks, Wildlife and Plant Conservation (DNP), and the National Research Council of Thailand (NRCT) under a benefit‐sharing agreement (Permit Numbers: 0002.3/2647, 0002/17, 0002/4137, 0402/2798, 0401/11121). The study was purely observational and followed the American Society of Primatologists (ASP) principles for the ethical treatment of nonhuman primates, as well as the American Society of Primatologists Code of Best Practices for Field Primatology.

### 2.2 Study Site and Subjects

The study was carried out on a population of Assamese macaques at the study site Phu Khieu Wildlife Sanctuary, Thailand that has been followed since 2006. The study area consists of hill evergreen forest with patches of bamboo forest with large populations of large herbivores and predators, which indicates its low level of disturbance (Borries et al. 2011). Reproduction is seasonal with ~80% of births occurring in April–June and most conceptions between November and January (Fürtbauer et al. 2010; Shivani et al. 2025; Touitou et al. 2021). Despite seasonal reproduction, environmental predictability is low, with high variation in fruit availability within and between years (Berghänel et al. 2016; Heesen et al. 2013). Infancy was defined as the first 12 months of life, given that females can give birth to a new offspring the year after the last offspring was born (Shivani et al. 2025) and nipple contact is observed during the first year of age, despite a significant reduction around 6 months of infant age (Berghänel et al. 2016).

In this study, we focused on 59 infants born in three cohorts (2011, 2018, 2022) to 40 different mothers, with a sex distribution of 33 male (56%) and 26 female (44%) infants. Of the 40 mothers, 15 mothers had offspring in more than one cohort (12 in two cohorts, 3 in three), and 10 of the infants had primiparous mothers (17%). Individuals were identified based on physical features, and mother–offspring relatedness was determined through observations of nipple contact during the infant's first weeks of life. This method has been validated using microsatellite markers in an earlier study on 43 infants, in which likelihood analyses confirmed all maternity assignments based on behavioral observations (Sukmak et al. 2014). Additionally, across 20 years of observation, we found no evidence of allomaternal nursing in the population. Of the 59 infants, all but 4 female infants survived until the end of their first year of life. These female infants were included in the analysis since they disappeared late (at 5 and 7 months of age), but one male infant that was 1 month old when disappeared was excluded from the analysis. We had nine combinations of group and year in our data, which resulted from various splits of the original groups across years. In 2011, one study group was followed, in 2018 three, and in 2022 five. Between‐group variation in size ranged from 18 to 74 individuals, but within‐group variation per year was relatively low (*mean* = 8.875, sd = 2.9).

### 2.3 Data Collection

To record mother–infant interactions, we conducted 40‐min focal animal observations on infants from birth to the end of infancy (up to 1 year of age), combining continuous recording of behaviors and instantaneous sampling every 2 min (Altmann 1974). Based on the long‐term project's ethogram and considering the variables commonly used to describe mother–infant relationships in primates (e.g., Arbaiza‐Bayona et al. 2022; Bădescu et al. 2022; Bardi and Huffman 2002; Castellano‐Navarro et al. 2023; Czerwinski et al. 2025; Deng and Zhao 1991; Dittus and Baker 2024; Revathe et al. 2024; Roura‐Torres et al. 2025; Stead et al. 2025), we continuously recorded the time and direction of initiation and termination of body contact and proximity (within 1.5 m) between infants and mothers, as well as events in which the mother restrained her infant's movement or refused the infant's attempts to establish body or nipple contact (by pushing the infant away, moving the nipple away by hand, or turning away). We were able to record the initiation and termination of mother–infant body contact and proximity in 55% and 63% of all cases, respectively. In the instantaneous part of the protocol, we recorded whether the infant was in nipple contact with the mother, being carried by the mother, or moving or feeding independently (see Table S1 for the definition of each behavior). As data were collected by two or more observers during each data collection period, we regularly conducted parallel protocols to ensure interobserver reliability.

In 2011, one group was continuously followed, while in 2018 and 2022, each group was followed during 5–10 consecutive days each month. We aggregated the data per 15‐day periods, with 365.23 days of age divided in 25 periods. This resulted in 3–22 data points for each mother–infant dyad (*mean* = 12.13, sd = 4.64), depending on the observation effort and survival of the infant. Our total observation time was 2214 h, with a mean observation time per data point of 3.09 h (sd = 1.72 h). This represented 70,098 sampling points of the instantaneous sampling, with a mean number of sampling points per data point of 97.9 (sd = 54.39). A detailed description of the observation effort for each mother–infant dyad can be found in the Supporting Material (Figure S1 and Table S2).

### 2.4 Model Formulation

#### 2.4.1 Infant Age

2.4.1.1

The age of the infant was included in the model as the main predictor of maternal and infant behavior. The day of birth of the infant was either known to the exact day or was calculated as the midpoint between the last day the mother was seen without an infant and the first day the infant was seen. Exact date of birth was known for 23 of the 59 infants; for the remaining infants the median error around the date of birth was 12.29 days (Q1 = 2 days, Q3 = 16 days, range = 2–24). Given that we aggregated the data into 15‐day periods, we used the mean infant age (in days) of all observations within each period.

Based on our expectations regarding the age‐related trajectories of the mother–infant spatial relationship and the infant's transition from dependent to independent feeding and locomotion, we predicted a nonlinear relationship between age and both infant and maternal behaviors, which we modeled using a Gompertz function (Gompertz 1825). The Gompertz function is a sigmoid function, which unlike the logistic function, allows for an asymmetric approach to the curve's initial and final asymptotes. In addition, other than a standard logistic model, it allows for a lower asymptote larger than 0 and an upper asymptote smaller than 1. Although the Gompertz function was originally developed to explain changes in mortality rates as a function of human age (Gompertz 1825), it has been applied to model temporal changes in other biological processes, such as body growth in mammals (Zullinger et al. 1984). Also, the Gompertz function has been used to fit primate data on the ontogenetic trajectory of time spent in suckling and out of contact with caretakers (Tab Rasmussen and Tan 1992).

We modified the original function by adding a constant representing the initial or left asymptote (*d*):

(1) $$y(x)=d+a\cdot e^{-b\cdot e^{-c\cdot x}},$$

where *y* is the variable representing maternal or infant behavior, *d* is the initial or left asymptote, *a* is the amplitude of change, determining the difference between *d* and the final or right asymptote of *y*(*x*), *b* determines the displacement along the time axis, together with *c* controlling at which value of *x* the steepest change of *y* occurs, *e* is Euler's number (base of the natural exponential), *c* is the rate parameter that controls how quickly *y*(*x*) transitions between its asymptotes, and *x* represents infant age, the independent variable driving changes in *y*(*x*).

This function models the increase in infant independence (*y*) with respect to infant age (*x*) (i.e., *a* > 0), whereas a decrease with age was expected for maternal care (i.e., *a* < 0), and hence instead of adding (*a*) to (*d*), we subtracted (*a*) from (*d*).

#### 2.4.2 Fruit Availability

Since Assamese macaques reproduce seasonally, we wanted to control for environmental conditions that vary with offspring age and could also influence infant and maternal behavior. Specifically, given that Assamese macaques in this population are predominantly frugivorous (Schülke et al. 2011), we included an index of population‐level fruit availability as a potential confounding variable in our models, which has been shown to correlate with female energy intake, behavioral changes, and hormonal levels (Heesen et al. 2013). Fruit abundance was measured at the middle of every calendar month in several individuals of 30 tree species that in more than 1 month accounted for more than 5% of the population's monthly fruit feeding time and collectively attracted more than 72% of the population's total fruit feeding time over a 10‐year study period. For each tree species *i,* the monthly index was calculated by combining (a) the average abundance of fruits across the monitored individuals of the species (*Ai*), (b) the estimated density of the species over 20.25 ha of botanical plots within the study area (*Di*), and (c) the average basal area of the species (*Bi*). The resulting values for the 30 species (*n*) were summed to calculate the monthly Fruit Availability Index (FAI) as Equation (2):

(2) $$\text{FAI}=\sum_{i}^{n}\left(A_{i}\cdot D_{i}\cdot B_{i}\right).$$

Fruit abundance was assessed at mid‐month. Therefore, we linearly interpolated daily FAI values between consecutive months to generate a smooth, continuous estimate of fruit availability throughout the year. This approach allowed us to calculate, for each data point (corresponding to an infant 15‐day period), a mean FAI value representing the fruit availability during the specific date range of that period, regardless of its position within a given month. Fruit availability showed considerable variation between and within years in the population (*mean* = 15.32, sd = 9.75), and varied substantially between our study years (2011: *mean* = 42.2, sd = 16, 2018: *mean* = 30.2, sd = 17.2, 2022: *mean* = 21.4, sd = 11.8).

#### 2.4.3 Maternal Characteristics

Maternal parity, maternal age, and dominance rank can lead to variations in maternal care among females (Altmann and Samuels 1992; Bădescu et al. 2022; Berman et al. 1994; Liu et al. 2024; Roura‐Torres et al. 2025; Soben et al. 2023; Tanaka 1989). We did not aim to explain the effects of these variables on the mother–infant relationship, but still initially included maternal rank, maternal age, and parity as additional fixed effects to account for biases arising from unbalanced sampling (e.g., we could not choose which mothers gave birth in the studied cohorts) and to improve model precision. We assumed that there was no true causal relationship between parity, mother's age, and maternal rank and any of the other variables included in the model.

Although maternal parity and age are correlated for primiparous and young mothers, both were initially included in the models because they influence maternal care behavior through different mechanisms (maternal experience vs. maternal condition, Fairbanks 1996). We did not use the number of birth events per female, but only differentiated primiparous from multiparous mothers, as additional births are unlikely to produce substantial behavioral differences. Due to collinearity between maternal age and parity, expressed by high variance inflation factor, we excluded maternal age from our final models.

To estimate the dominance rank of the 40 mothers, we applied the Elo‐rating method (Albers and De Vries 2001) using the elo.seq function from the EloRating R package (Neumann and Kulik 2024) with 1000 as the starting value and 100 as the gain constant k (De Moor et al. 2020; Stranks et al. 2024). Data points were decided dyadic conflicts between females during which the losing female exhibited only submissive behaviors (bared‐teeth display, make‐room, give‐ground), while the winner showed no submissive behaviors (Hausfater 1974). The winner either displayed only aggressive behaviors (open‐mouth threat, lunge, slap, bite, chase) or did not actively provoke the submission. The Elo‐rating calculations for each group included the history of wins and losses from all recorded conflicts. Thus, although Elo scores were calculated based on the individuals present in each group at a given moment, they were still influenced by the history of agonistic events prior to the group split. We used standardized Elo‐scores (assigning 1 to the highest and 0 to the lowest score per group, see Table S3) at the beginning of each birth season to represent the relative rank of each mother within the group.

#### 2.4.4 Data Analysis

We excluded data points for which we had < 1 h of observation time for an infant in a given period to aim for representative data on infant and mother behavior. This left 716 data points in total. However, models using instantaneous sampling data included 715 data points because of missing data for one combination of period and infant. Also, for some models in which the response variable was a quotient, data points were excluded when the total number of occurrences in the denominator was 0 (e.g., no proximity changes), reducing the total number of data points used to fit the model (see Table 1).

**Table 1:** Infant and mother measures analyzed.

Infant or mother behavior	Definition	Error distribution	*N*
Mother–infant spatial relationship			
Proportion of time in proximity	Time the infant spends in proximity (i.e., within 1.5 m) of its mother as a proportion of the total observation time.	Beta	716
Proportion of proximity initiations	Number of proximity (i.e., within 1.5 m) initiations made by the infant or the mother (approaches), relative to all proximity changes (approaches and departs) initiated by the same individual.	Binomial	Mother: 369 Infant: 494
Responsibility in maintaining proximity	Proportion of proximity initiations (within 1.5 m) by the mother toward the infant, relative to the total proximity initiations by both members of the dyad, minus the proportion of proximity terminations by the mother, divided by the total proximity terminations by both members of the dyad. The response was then rescaled to range from 0 to 1, whereby higher values indicate greater responsibility of the mother in maintaining proximity. Values above 0.5 reflect more responsibility of the mother compared to the infant.	Beta	595
Proportion of time in body contact	Time the infant spends in body contact with its mother as a proportion of the total observation time.	Beta	716
Proportion of body contact initiation	Number of body contact initiations made by the infant or the mother, relative to all body contact changes (initiations and terminations) initiated by the same individual.	Binomial	Mother: 275 Infant: 465
Responsibility in maintaining body contact	Proportion of body contact initiations by the mother toward the infant, relative to the total body contact initiations by both members of the dyad, minus the proportion of body contact terminations by the mother, relative to the total body contact terminations by both members of the dyad. The response was then rescaled to range from 0 to 1, whereby higher values indicate greater responsibility of the mother in maintaining body contact. Values above 0.5 reflect more responsibility of the mother compared to the infant.	Beta	593
Rate of restraints	Rate of maternal restraining events per hour of observation time.	Descriptive data	716
Transition from dependent to independent feeding and locomotion			
Proportion of time in nipple contact	Number of instantaneous sampling points in which the infant is in nipple contact, divided by the total number of instantaneous sampling points.	Beta	715
Rate of mother refusing infant body or nipple contact initiation	Rate of mother refusing the infant's attempts to establish body or nipple contact per hour of observation time.	Descriptive data	716
Proportion of time of independent feeding	Number of instantaneous sampling points in which the infant is feeding independently, divided by the total number of instantaneous sampling points.	Beta	715
Proportion of time carrying	Number of instantaneous sampling points in which the infant is carried by the mother, divided by the total number of instantaneous sampling points.	Beta	715
Proportion of time in independent locomotion	Number of instantaneous sampling points in which the infant is locomoting independently, divided by the total number of instantaneous sampling points.	Beta	715

To describe the age‐trajectories of the mother–infant spatial relationship and the transition from dependent to independent feeding and locomotion, we extracted rates, proportions, or indices based on the infant and mother behaviors (Table 1). These measures have been traditionally used in the literature to study mother–infant relationships (e.g., Arbaiza‐Bayona et al. 2022; Bădescu et al. 2022; Bardi and Huffman 2002; Castellano‐Navarro et al. 2023; Czerwinski et al. 2025; Deng and Zhao 1991; Dittus and Baker 2024; Revathe et al. 2024; Roura‐Torres et al. 2025; Stead et al. 2025) and constituted the response variables in the fitted models. Given the scarcity of events observed during the data collection period, we could not fit models for the rate of restraints and for the rate of mothers refusing infant body or nipple contact initiation, but we provide descriptive statistics for these two variables.

We fitted a series of nonlinear generalized mixed models using the Bayesian software package brms (version 2.22.0, Bürkner 2025) in R version 4.5.1 (R Core Team 2025). These models incorporated a binomial error distribution for discrete proportions and a beta error structure for continuous proportions, both combined with an identity link function (see Table 1). The models included the Gompertz as described above. To estimate the parameters *c* and *b* in an unconstrained space while constraining their effective parameters to be positive (as they represent scaling factors that cannot be negative), we exponentiated them in the model fitting function. Similarly, the effective parameters a and d needed to be constrained within the range between 0 and 1, but we wanted to estimate them in an unconstrained space. Hence, we applied an inverse logit transformation in the model fitting function (e.g., $d_{\mathrm{transformed}}=\frac{e^{d}}{1+e^{d}}$). Additionally, to ensured that *d* + *a* does not exceed 1, since each response variable is bound between 0 and 1, we multiplied the effective value of a by the effective value of *d*. Thus, the amplitude of change was smaller than the initial asymptote ensuring that the final asymptote was larger than or equal to 0. Two exceptions were the models for the proportion of independent locomotion and feeding time, for which the amplitude of change was larger than the initial asymptote, and hence we multiplied the effective value of a by 1 − effective value of *d*. Constraining the effective parameters *d* + *a* ensured that the fitted model bound remained between 0 and 1, enabling us to fit all the models with identity link function.

Additionally, we included fixed effects for fruit availability, maternal parity, and maternal dominance rank, as described above. To simplify the interpretation of the model estimates, fruit availability was standardized using *z*‐scores (with a mean of 0 and a standard deviation of 1) (Schielzeth 2010). Parity and infant sex were added as binary dummy‐coded categorical variables. For infant sex, “female” was designated as the reference level (intercept). For parity, “multiparous” was defined as the reference level. Infant sex was initially included as an interacting term with infant age, by adding a coefficient for infant sex to each parameter of the Gompertz function described in Equation 1 (*ds*, *as*, *bs*, and *cs*). The fixed part of the model formula implemented in brms describing the relationship between the mother and infant behaviors (*y*) and infant age (*x*) is provided in the Supporting Material (Equation S1).

Since no differences across age were found between sexes (Figures S2–S4), we reduced the models by only adding independent effects for infant sex and age. Given that we had repeated measurements for infant, mother, and group ID, both initial and reduced models included them as random intercepts. Age and fruit availability varied within each mother, infant, and group ID, so these variables were included as random slopes within each grouping factor (Schielzeth and Forstmeier 2009). We included a random slope for each of the four parameters of infant age (*ds*, *as*, *bs*, and *cs*) within all three grouping factors and for each interactive term of infant age and sex (*ds*, *as*, *bs*, and *cs*) within mother ID and group ID. Finally, for group ID, we specified infant sex, maternal parity, and maternal rank as additional random slopes.

We chose weakly informative and normally distributed priors for the fixed effects (Table S4). In the case of the parameters of the Gompertz function, we chose their means based on a visual inspection of the data, with a standard deviation of 1 for parameters *a*, *c*, and *d*, and a standard deviation of 2 for parameter *b*. For all other fixed effects, we chose weak priors, namely a normal distribution with a mean of 0 and a standard deviation of 2. For the prior distributions of standard deviations of the random effects, we used weakly regularizing exponential priors with a rate of 1. We ran 10,000 iterations for each of 8 chains in the MCMC sampling process, with an adapt_delta parameter set to 0.85 or 0.99 when divergent transitions were present. The Effective Sample Size (ESS) values for Bulk ESS and Tail ESS were > 3000 for all models, indicating adequate sampling. Exceptions were the models for the mother's responsibility for maintaining body contact, and the proportion of carrying time, in which Bulk ESS and Tail ESS values were higher than 1000. Additionally, all models showed R‐hat values of 1, indicating no convergence issues, except for the mother's responsibility for maintaining body contact, which reached up to 1.01.

For each model, we specified the mean of the priors as the starting values for the parameters to provide stable initialization for the MCMC chains. Posterior predictive checks perform with the Bayesplot R package (Gabry et al. 2019) further suggested that the function was appropriate in capturing the age trajectory for most of the variables analyzed, although predicted values were far from observed values for some age ranges (Figure S5). Specifically, the model did not adequately fit the response variable for certain variables, such as the mother's responsibility in maintaining body contact and proximity.

To extract the models' predictions with regard to age and to quantify the uncertainty in them, we determined fitted values with respect to infant age for all posterior samples of model parameters. These posterior distributions of fitted values were then used to determine the posterior median and its 2.5% and 97.5% credible interval, respectively, of the fitted model. Posterior medians instead of means were used, as they are more robust in the presence of skewed distributions (Gelman et al. 2013). Additionally, for each model, we report the location of the inflection point ($\frac{\log\left(b\right)}{c}$) the initial asymptote (*d*), and the final asymptote (*a*), all expressed on the natural scale after back‐transforming them to their constrained space.

Given that the fitted nonlinear trajectories depend on four parameters describing age‐related changes, sex estimates from our models necessarily reflect differences while these age parameters were held constant (at the mean age of the sample). To better interpret such differences, we additionally examined predicted values at the model's age of inflection point (i.e., when the rate of behavioral change is maximal).

Additionally, using the Bayesplot R package (Gabry et al. 2019), we created uncertainty plots that display the median and 95% credible intervals of the random effects, extracted from the posterior samples of each model. The models revealed substantial variation (i.e., relatively large estimated standard deviations for the random intercepts and slopes) as compared to the magnitude of the fixed effects estimates, but the estimated standard deviations were highly uncertain, as indicated by the wide credible intervals (Figures S6–S8). To further investigate the consistency of maternal behavior across mothers (Fairbanks 1996), we visually compared mothers' behavior based on (a) the posterior distributions of their median deviation from the population average (Figure S9), and (b) their individual fitted values across infant age (see Figure S10). Only for some mothers, the posterior distribution of their median deviations was consistently below or above the population average (Figure S9). Also, although for most mothers median predicted values were consistently above or below the population average across infant age, and in some cases the deviation from the population average was consistent between different infants of the same mother, confidence intervals overlapped, indicating a high degree of uncertainty in the estimated mother‐specific trajectories (Figure S10). This was expected given the increasing evidence of lack of consistency in maternal behavior across infant development (Czerwinski et al. 2025; Revathe et al. 2025). Therefore, offspring age should be included when evaluating maternal care consistency, particularly when sampling is uneven or incomplete (Mundry et al. 2023). As an analysis of consistency of maternal behavior was not an aim of our study, we provide one representative example of those plots only (Figures S9 and S10).

## 3 Results

### 3.1 Effect of Infant Sex on the Mother–Infant Relationship

**Figure 1:**
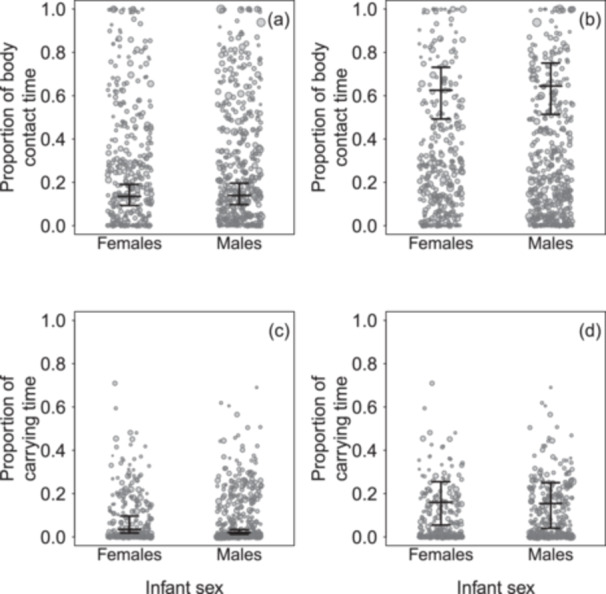
Effect of sex on the proportion of body contact time at (a) mean age (182.29 days) and (b) age inflection point (58.35 days), and on the proportion of carrying time at (c) mean age (182.05 days) and (d) age at inflection point (86.79 days). Dots represent infant‐period data points, with the area of each dot proportional to the observation effort for that data point (min = 1.00 h, max = 12.37 h). The horizontal lines indicate the median (50th percentile) of the posterior samples, while the vertical lines represent the 95% credible intervals.

The credible intervals of the coefficients of infant sex in most of the reduced models did not include 0, but were very wide (Tables S5 and S6). We visually explored potential sex differences for models in which the lower bound of the credible intervals was higher than 1 (proportion of contact time and proportion of carrying time), which also suggested no notable differences in the response variables concerning infant sex at mean age and age at the inflection point (Figure 1). Consequently, we do not present sex‐differentiated results in the subsequent analyses.

### 3.2 Age‐Trajectory of the Mother–Infant Spatial Relationship

**Figure 2:**
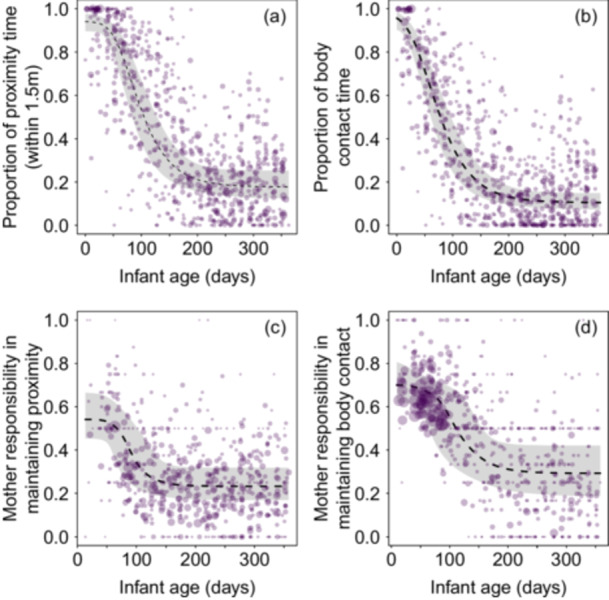
Effect of infant age on the proportion of time in mother–infant (a) proximity (within 1.5 m) and (b) body contact, and the mothers' responsibility in maintaining (c) proximity (within 1.5 m) and (d) body contact. Dots represent infant‐period data points, with the area of each dot proportional to the observation effort for that data point (proportion of proximity and body contact time: min = 1.00 h, max = 12.37 h; mother's responsibility in maintaining proximity and body contact: min = 2, max = 295). The dashed lines represent the median (50th percentile) of the posterior samples at each age value, while the shaded regions correspond to the 95% credible interval of the model predictions, determined as the 2.5th and 97.5th percentiles of the posterior samples.

During the first weeks of an infant's life, mothers spent the majority of the observation time in proximity (within 1.5 m; Figure 2a) and body contact (Figure 2b) with their infants. Both responses followed a decrease during the subsequent months with the inflection point occurring around 60–120 age‐days. Starting from month 7 (around 210 age‐days), the predicted proximity and body contact time fell below 25% (Table 2).

**Table 2:** Inflection point and initial and final asymptote for mother–infant spatial relationship models.

Model	Inflection point [95% CI]	Initial asymptote [95% CI]	Final asymptote [95% CI]
Proportion of proximity time	71.19 age‐days [55.38, 91.18]	0.94 [0.91, 0.97]	0.17 [0.08, 0.28]
Proportion of body contact time	58.36 age‐days [43.67, 74.27]	0.98 [0.91, 1.0]	0.11 [0.07, 0.16]
Mother responsibility in maintaining proximity	83.78 age‐days [52.90, 123.50]	0.54 [0.45, 0.66]	0.26 [0.08, 0.51]
Mother responsibility in maintaining body contact	78.70 age‐days [32.50, 125.59]	0.72 [0.62, 0.83]	0.31 [0.08, 0.76]
Proportion of mother proximity initiation	64.05 age‐days [36.52, 84.68]	0.56 [0.48, 0.68]	0.34 [0.24, 0.50]
Proportion of infant proximity initiation	61.08 age‐days [24.58, 91.29]	0.51 [0.43, 0.57]	0.72 [0.66, 0.77]
Proportion of mother body contact initiation	92.18 age‐days [74.21, 110.46]	0.85 [0.80, 0.90]	0.38 [0.24, 0.54]
Proportion of infant body contact initiation	106.27 age‐days [71.30, 140.22]	0.40 [0.36, 0.42]	0.69 [0.61, 0.76]

Mothers were primarily responsible for maintaining body contact and proximity with their offspring during the first months of infancy (Figure 2c,d), with initial asymptotes above 0.5 (see Table 2). This responsibility shifted to the infant when it reached 2–4 months of age (around 60–120 age‐days).

**Figure 3:**
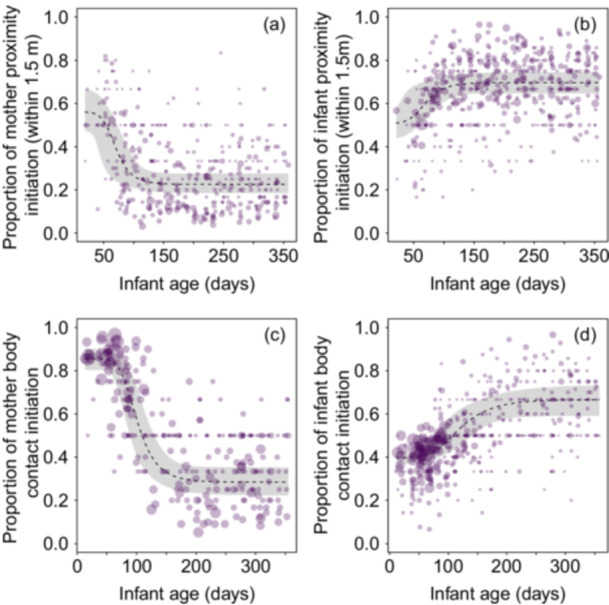
Effect of infant age on the proportion of (a) mother proximity initiation, (b) infant proximity initiation, (c) mother body contact initiation, and (d) infant body contact initiation. Proportions are calculated relative to all proximity or body contact changes initiated by the same member of the dyad. Dots represent infant‐period data points, with the area of each dot proportional to the observation effort for that data point (min = 2, max = 236). The dashed line represents the median (50th percentile) of the posterior samples at each age value, while the shaded region corresponds to the 95% credible interval of the model predictions, determined as the 2.5th and 97.5th percentiles of the posterior samples.

The number and area of the dots in Figure 3a,b indicate that at the beginning of infancy, there were very few changes in proximity between mothers and infants, whereas the opposite was observed for body contact initiation (Figure 3c,d). During the initial days, mothers initiated more interactions than they terminated, both in terms of proximity and body contact (Table 2 and Figure 3a,c). However, this pattern gradually shifted, with mothers terminating more interactions than they initiated as the infant grew older. As shown in Figure 3a,c, and according to the inflection points (Table 2), the shift also occurred around 60 days of age.

The pattern described above was reversed for the proportion of infant body contact initiation, which increased by about 20% from the beginning to the end of infancy (Figure 3d and Table 2). In this case, fitted values were above 0.5 from month 3 (around 90 days of age). Although the proportion of infant proximity initiation also increased from the beginning of infancy to the end (Figure 3b and Table 2), infants consistently approached their mothers more frequently than they left them throughout infancy.

Restraining events were registered only in 21.11% of the data points (*n* = 649 events). The time window of restraining started with an infant of 2.5 days of age and ended at 294 days of age. For the subset of data points in which restraint occurred, the mean age was 67.46 days (sd = 43.60). When considering all data points in which mothers had the opportunity to restrain their infants (by being in body contact with them), the average restraining rate was 0.6 events per hour (sd = 1.6, min = 0, max = 15.72).

### 3.3 Age‐Trajectory of the Transition From Dependent to Independent Feeding and Locomotion

**Figure 4:**
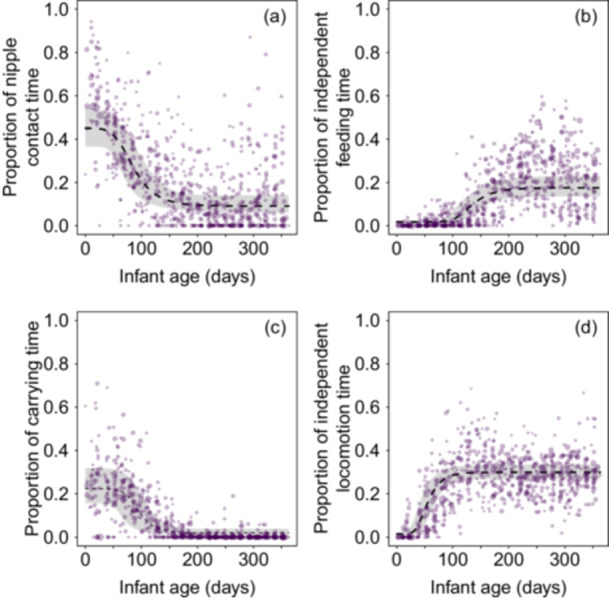
Effect of infant age on the proportion of time in (a) nipple contact, (b) carrying, (c) independent feeding, and (d) independent locomotion. Dots represent infant‐period data points, with the area of each dot proportional to the observation effort for that data point (min = 10 sampling points, max = 389 sampling points). The dashed lines represent the median (50th percentile) of the posterior samples at each age value, while the shaded regions correspond to the 95% credible interval of the model predictions, determined as the 2.5th and 97.5th percentiles of the posterior samples.

Nipple contact time accounted for around 40% of the observation time in newborns, gradually decreasing to < 10% by around 210 days of age (7 months), and varied considerably between individual observation periods (Figure 4a and Table 3). With respect to carrying time, mothers carried their newborns around 20% of the observation time, with fitted values remaining above 20% until around 60 days of age (2 months, Figure 4c and Table 3). By around 120 days of age (4 months), mothers carried infants for 10% or less of the observed time, and after 120 days of age (7 months), the majority of individual observation periods had no occurrence of maternal carrying, but carrying was still observed twice in an infant of 11.8 months of age.

**Table 3:** Inflection point and initial and final asymptote for the transition from dependent to independent feeding and locomotion models.

Model	Inflection point [95% CI]	Initial asymptote [95% CI]	Final asymptote [95% CI]
Proportion of nipple contact time	75.71 age‐days [51.51, 101.34]	0.45 [0.37, 0.55]	0.08 [0.05, 0.13]
Proportion of independent feeding time	125.0 age‐days [102.0, 145.3]	0.11 [0.07, 0.17]	0.30 [0.26, 0.34]
Proportion of carrying time	89.05 age‐days [52.34, 109.26]	0.22 [0.15, 0.31]	0.02 [0.01, 0.03]
Proportion of independent locomotion time	128.45 age‐days [100.15, 153.15]	0.01 [0.00, 0.02]	0.33 [0.28, 0.38]

Infants rarely fed (Figure 4b and Table 3) or locomoted (Figure 4d and Table 3) independently of their mothers during their first month of life, after which they steadily increased their proportion of independent locomotion time until around 90 days of age (3 months). In the case of independent feeding, a steady increase was observed around 90 days of age (3 months) until around 210 days of age (7 months).

We observed a total of 340 events of mothers refusing infant body or nipple contact initiation (in 47.8% of the individual observation periods), distributed across almost all infancy (first case observed at 67 days of age and last case at 356.83 days of age). The mean age for the subset of the data which comprised the observed cases of mothers' refusal was 224.74 days of age (sd = 79.58). When mothers had the opportunity to reject their infants (by being in spatial proximity with them), mothers refused their infants an average of 0.79 times per hour (sd = 2.43, min = 0, max = 28.8).

## 4 Discussion

Our findings align with the age‐trajectory of mother–infant relationships commonly described in primates (Bădescu et al. 2016; Förster and Cords 2002; Revathe et al. 2024) and specifically macaques (Berman 1980; Castellano‐Navarro et al. 2023; Dittus and Baker 2024; Lindburg 1971), with mothers expected to gradually decrease their care over time, and the steepest reduction predicted for the early months as infants begin to show increasing independence. At birth, infants of the study population almost fully depended on their mothers, who substantially invested in their offspring by feeding and carrying them. A transitional phase emerged early, between 1 and 3 months of age, marked by a noticeable reduction in maternal care. The reduction in maternal care coincided with a significant increase in independent locomotion and with the start of independent feeding by infants. By 6–7 months of infant age, the rate of decrease in maternal care and the rate of increase in infant independence had slowed down. Specifically, body contact and proximity between mothers and infants had decreased substantially (from 100% to < 50% of the observed time in most data points), and infants had achieved near‐complete independence in locomotion. Although nipple contact persisted (as observed in previous studies; Berghänel et al. 2016), infants spent nearly twice as much time feeding independently. Finally, contrary to our expectations, the age‐trajectory and the average levels of maternal care and infant independence across infancy did not change depending on the infant sex, although the high uncertainty of the sex effect in all the models, probably related to the limited sample size of the study, warrants cautious interpretation. The lack of sex differences might suggest, however, that such differences depend on other factors, such as mother age (Soben et al. 2023), or that they emerge later in development, particularly during the juvenile period (Kulik et al. 2016). The later explanation corresponds with the increasing sex differences in body size observed with increasing individual age in our population (Anzà et al. 2022).

Similar to other macaque species (Berman 1980; Dittus and Baker 2024; Lindburg 1971), by the second half of the first year of the infant's life, maternal care was significantly reduced and correspondingly infants were highly independent. As commonly observed in primates (Fairbanks 1996; R. A. Hinde and Spencer‐Booth 1967; Lindburg 1971), the push toward infant independence was largely initiated by mothers, with infants adapting accordingly. During the initial months, infants were highly active, frequently breaking physical contact with their mothers to explore their surroundings. Mothers, in turn, actively sought to maintain contact, often restraining their infants' attempts to move away. However, in the transitional phase, mothers began to refuse contact attempts and increased spatial distance from their offspring, while infants responded by intensifying efforts to maintain proximity and re‐establish contact. This pattern led to older infants being mainly responsible for the remaining spatial proximity with their mothers.

As in other primates, locomotor independence preceded feeding independence: a gap of around 2 months with respect to the first signs of independence, and a larger gap in the full achievement of independence (carrying ended around 6 months of age, while nipple contact persisted until the end of infancy). Earlier cessation of carrying compared to nursing has been explained by the higher energetic costs of the former (Altmann and Samuels 1992; Young and Shapiro 2018). The shift around 2 months of age for the transition from maternal carrying to independent locomotion described in our population, aligns with the acquisition of several motor skills in the population during this phase (Berghänel et al. 2015). Also, the timing of this transition resembles that reported for wild toque (*Macaca sinica* [*M. sinica*], Dittus and Baker 2024) and free‐ranging rhesus macaques (Lindburg 1971), while being significantly earlier than in non‐macaque species of similar size. For example, Geoffroy spider monkeys shift from maternal carrying to independent locomotion only at the end of the first year of age (Arbaiza‐Bayona et al. 2022). Since body and brain size are similar in the two genera, the difference in the timing of locomotion independence is most probably explained by their phylogenetic distance. Other interspecific differences, such as the degree of arboreality and diet, have not been linked to variation in the timing of independent locomotion (Young and Shapiro 2018). To clarify the mother–infant dynamics underlying the early locomotor independence observed in our Assamese macaque population, it will be important to distinguish maternal carrying from maternal rejection of nipple contact.

Regarding the other milestone in mammalian infant independence, age of weaning, two events must be considered: the end of the mother's milk provision, and the first day in which infants eat solid food (Langer 2003). Our infants began to consume solid food as early as 2 months, which is consistent with the mean age reported across 16 cercopithecid species (Langer 2003), and with observations from other macaque populations (Dittus and Baker 2024). The start of solid food consumption was accompanied by a significant decrease in nipple contact time during the first half of infancy, coinciding with a peak in maternal rejection at 7 months of age that resembled that of other wild macaques (*M. sinica*, Dittus and Baker 2024); *M. silenus*, Krishna et al. 2008; *thibetana*, Deng and Zhao 1991). It also aligned with a reported average end of lactation in cercopithecines at around 7 months of age (Langer 2003). However, the infants of our population stayed in nipple contact with their mothers ~10% of the observed time well into the second half of their first year of life. Milk consumption is difficult to estimate from observation only (K. Hinde 2009), given that it is often methodologically unfeasible to distinguish between suckling and nipple contact in wild, arboreal populations. Thus, we would need to directly measure the presence or absence of milk consumption to be able to conclude the exact age in which immature Assamese macaques become nutritionally independent of their mothers (for instance, with stable isotope analysis: Crowley and Hinde 2012). Notably, by directly assessing the mammary tissue of wild toque macaque mothers, Dittus and Baker (2024) reported that while some infants stopped accessing maternal milk by 7.2 months, most have available milk until 18 months or later. This suggests that female macaques may employ a mixed feeding strategy to optimize both infant survival and maternal reproductive success. Accordingly, the persistence of nipple contact likely reflects extended energetic maternal support rather than merely comfort, as proposed by some studies (e.g., Bădescu et al. 2016). Nonetheless, the high interindividual variation and the lack of information on nocturnal suckling demand further investigation of the weaning transition in this population.

Our results provide evidence for behavioral adaptations in maternal care that allow Assamese macaque females to maintain their reproductive schedules in accordance with the seasonal ecological conditions of their natural environment. Assamese macaques follow a relaxed income breeding strategy (Heesen et al. 2013; Touitou et al. 2021), combining elements of both income and capital breeding. Consistent with an income breeding strategy, the period of peak lactation is on average coinciding with the peak food abundance, yet the link between physical condition and conception probability is an element of capital breeding (Heesen et al. 2013). This pattern, along with our previous findings of an occasional 1‐year interbirth interval predicted by the age of the previous offspring (Shivani et al. 2025) sets the stage for a mother–offspring conflict over energy allocation. While it may theoretically be possible for a mother to strategically allocate maternal resources depending on the food availability experienced in a current birth season, in reality, food availability is highly unpredictable between and within years, and the birth season lasts several months (Heesen et al. 2013). Consequently, females giving birth in the same year experience vastly different conditions during their lactation period (Anzà et al. 2025), hampering fine‐scaled strategic adjustment of maternal care. Instead in a highly unpredictable environment as at the study site, it may be the best strategy for a mother and her infant to fast‐track infant independence and to promote a prolonged mixed feeding strategy consisting of milk and independently foraged solid food (Dittus and Baker 2024; Langer 2003), as we observe in our population.

An additional piece of evidence for the adjustment of Assamese macaque infants to the reproductive pace of their mothers comes from a study on behavioral responses of immatures to the birth of a new sibling (Pavlovic et al. unpublished manuscript). The transition to siblinghood often marks a period of intense mother–offspring conflict as resources are allocated away from the current offspring into the next (Volling 2012). In line with this conflict, immature primates have been shown to react to the birth of a sibling by increased behavioral distress (chacma baboons [*P. ursinus*], Delaunay et al. 2023) and increases in glucocorticoid levels (bonobos, Behringer et al. 2022), and to increased mother–offspring competition due to maternal reproductive timing with increases in tantrums (*P. ursinus*; Dezeure et al. 2021). Comparing spatial and social data of Assamese immatures and their mothers between the months before and after the birth of a new sibling, mothers reduced their time in proximity with their older immature upon the arrival of a new infant creating the potential for conflict. However, immatures did not compensate for the reduction in maternal attention which suggest no evidence for a pronounced parent‐offspring conflict or sibling rivalry in the study population (Pavlovic et al. unpublished manuscript).

Collectively, this study provides a quantitative description of age‐related changes in the mother–infant relationship in a wild population of Assamese macaques. The observed transition toward independence aligns with the relaxed income‐breeding strategy proposed for the population (Heesen et al. 2013; Touitou et al. 2021), in which mothers reduce investment during late infancy and reallocate energy toward future offspring. Consequently, infants became primarily responsible for maintaining interactions with their mothers, who continue to provide limited support.

Modeling these changes as nonlinear trajectories captures the ontogenetic trajectories of the development of mother–infant relationships that linear approaches often overlook or neglect. The Gompertz function offered biologically interpretable parameters and avoided overfitting, though future studies may benefit from exploring alternative nonlinear functions, such as other sigmoidal models (Zullinger et al. 1984), to improve model fit and parameter interpretation. The extent to which the shape of the nonlinear trajectories is moderated by additional predictors warrants future analyses.

By establishing a quantitative baseline for nonlinear developmental trajectories in the mother–infant relationship, this study provides a framework for identifying when key transitions occur and how rapidly they unfold. Future work could evaluate whether inflection points in weaning, maternal carrying, or independent locomotion shift across individuals differing in rank, body condition, or experience, and across groups or populations differing in resource availability, group size, or social structure. Linking these nonlinear developmental profiles to individual, ecological, and social contexts will clarify whether variation in independence reflects plastic responses to environmental pressures or deeper life‐history differences across primates.

## Author Contributions

**Ana Lucia Arbaiza‐Bayona:** conceptualization, data curation, formal analysis, funding acquisition, investigation, methodology, visualization, writing – original draft. **Roger Mundry:** formal analysis, software, visualization, and writing – review and editing. **Suchinda Malaivijitnond:** project administration, writing – review and editing. **Suthirote Meesawat:** project administration, writing – review and editing. **Oliver Schülke:** conceptualization, methodology, funding acquisition, resources, project administration, supervision, and writing – review and editing. **Julia Ostner:** conceptualization, methodology, funding acquisition, resources, project administration, supervision, and writing – review and editing.

## Ethics Statement

The study was purely observational and followed the American Society of Primatologists (ASP) principles for the ethical treatment of nonhuman primates, as well as the American Society of Primatologists Code of Best Practices for Field Primatology.

## Supporting information

Supplementary Material.

## Data Availability

The data supporting the findings of this study are openly available in GRO.data, under the title “Replication data for: Age‐trajectory of mother–infant relationships in wild Assamese macaques.” The data set includes raw data and an example R script.
