# Supplementary material for: Age‐Trajectory of Mother–Infant Relationships in Wild Assamese Macaques
Source: Am J Primatol. 2026 Jan 20;88(1):e70110. doi: 10.1002/ajp.70110 (PMC12820445; doi:10.1002/ajp.70110)
Supplement: Supplementary file 1 — Supplementary Material. [file AJP-88-e70110-s001.docx]

**Supplementary material: Age-trajectory of mother-infant relationships in wild Assamese macaques**

Arbaiza-Bayona, Ana Lucia ^1, 2, 3^ ORCID: 0000-0002-8218-2551

Mundry, Roger ^3, 4, 5^ ORCID: 0009-0008-9988-5131

Malaivijitnond, Suchinda ^6, 7^ ORCID: 0000-0003-0897-2632

Meesawat, Suthirote ^6, 7^ ORCID: 0000-0001-8612-2436

Schülke, Oliver ^1, 2 ,3^ ORCID: 0000-0003-0028-9425

Ostner, Julia ^1, 2, 3^ ORCID: 0000-0001-6871-9976

^1^Department of Behavioral Ecology, Georg-August-Universität Göttingen, Johann-Friedrich-Blumenbach Institute for Zoology and Anthropology, Kellnerweg 6, D-37077 Göttingen, Germany.

^2^Research Group Primate Social Evolution, German Primate Center, Leibniz Institute for Primate Research, Kellnerweg 4, 37077 Göttingen, Germany.

^3^Leibniz ScienceCampus Primate Cognition, German Primate Center, Leibniz Institute for Primate Research, Kellnerweg 4, 37077 Göttingen, Germany.

^4^Department for Primate Cognition, Georg-August-Universität Göttingen, Johann-Friedrich-Blumenbach Institute, Kellnerweg 4, 37077 Göttingen, Germany.

^5^Cognitive Ethology Laboratory, German Primate Center, Leibniz Institute for Primate Research, Kellnerweg 4, 37077 Göttingen, Germany.

^6^National Primate Research Center of Thailand, Chulalongkorn University, Saraburi, Thailand.

^7^Department of Biology, Faculty of Science, Chulalongkorn University, Bangkok, Thailand.

**Corresponding author:** Ana Lucia Arbaiza-Bayona, a.arbaizabayona@stud.uni-goettingen.de

**Table S1.** Ethogram of mother infant behaviors

| **Behavior** | **Definition** | **Type of sampling** |
| --- | --- | --- |
| Proximity initiation | The mother or infant approaches the other member of the dyad within 1.5m (this behavior was always recorded even when the individual only passed by and moved swiftly in and out of the 1.5m sphere) | Continuous |
| Proximity termination | The mother or infant leaves a 1.5 m radius around the other member of the dyad | Continuous |
| Body contact initiation | The mother or infant comes so close to the other member of the dyad that parts of their bodies touch (physical contact during fighting was not recorded) | Continuous |
| Body contact termination | The mother or infant increases distance to the other member of the dyad, until no parts of their bodies touch anymore | Continuous |
| Mother restraints | The mother restrains the movement of the infant by holding its arm, leg, foot or tail. This was not coded as a state but as an event (i.e., recorded only once even if the behavior lasted a long period of time). It was recorded again every time the mother stopped holding the infant and started holding it again. | Continuous |
| Mother refuses infant body or nipple contact initiation | The infant tries to make body or nipple contact with the mother and the mother refuses to allow it by pushing the infant away, moving away her nipple with her hand or turning away. It was recorded again every time the mother refused contact, even if it was part of the same behavioral sequence. | Continuous |
| Nipple contact | The infant is in oral contact with the mother’s nipple. | Instantaneous |
| Independent feeding | Infant manipulates or ingests food that is not the mother’s milk. | Instantaneous |
| Carrying | The infant is on the mother while the mother moves, hangs or stands. | Instantaneous |
| Independent locomotion | The infant walks, runs, trots, jumps or climbs. | Instantaneous |

**Figure S1.** Observation effort of mother–infant dyads. Dots represent data points and dotted vertical lines defined the 15-day periods in which the first year of infancy was divided (25 in total).

**Table S2.** Descriptive summary of observation effort of mother–infant dyads

| **Infant ID** | **Total data points** | **Observation time** | | | **Instantaneous sampling points** | | |
| --- | --- | --- | --- | --- | --- | --- | --- |
|  |  | **Total** | ***m*** | ***sd*** | **Total** | ***m*** | ***sd*** |
| **Infant 1** | 7 | 19 | 2.77 | 1.85 | 594 | 84.86 | 55.37 |
| **Infant 2** | 13 | 49 | 3.76 | 1.82 | 1520 | 116.92 | 59.91 |
| **Infant 3** | 9 | 29 | 3.17 | 1.18 | 896 | 99.56 | 35.41 |
| **Infant 4** | 11 | 26 | 2.34 | 1.09 | 816 | 74.18 | 33.94 |
| **Infant 5** | 10 | 29 | 2.89 | 1.45 | 924 | 92.4 | 47.28 |
| **Infant 6** | 10 | 49 | 4.93 | 2.95 | 1559 | 155.9 | 93.03 |
| **Infant 7** | 7 | 34 | 4.91 | 3.65 | 1091 | 155.86 | 115.38 |
| **Infant 8** | 21 | 56 | 2.64 | 1.27 | 1774 | 84.48 | 42.6 |
| **Infant 9** | 8 | 15 | 1.83 | 0.87 | 469 | 58.62 | 27.38 |
| **Infant 10** | 19 | 48 | 2.54 | 1.22 | 1572 | 82.74 | 37.01 |
| **Infant 11** | 12 | 31 | 2.6 | 0.98 | 955 | 79.58 | 29.76 |
| **Infant 12** | 10 | 42 | 4.18 | 3.23 | 1337 | 133.7 | 100.58 |
| **Infant 13** | 18 | 49 | 2.71 | 1.29 | 1567 | 87.06 | 40.35 |
| **Infant 14** | 21 | 58 | 2.77 | 1.12 | 1893 | 90.14 | 36.15 |
| **Infant 15** | 12 | 39 | 3.25 | 1.67 | 1229 | 102.42 | 52.48 |
| **Infant 16** | 12 | 38 | 3.16 | 2.15 | 1188 | 99 | 68.26 |
| **Infant 17** | 9 | 24 | 2.64 | 1.36 | 758 | 84.22 | 43.42 |
| **Infant 18** | 9 | 41 | 4.57 | 1.65 | 1302 | 144.67 | 51.86 |
| **Infant 19** | 22 | 61 | 2.75 | 0.84 | 1989 | 90.41 | 28.37 |
| **Infant 20** | 3 | 12 | 4.11 | 1.2 | 390 | 130 | 38 |
| **Infant 21** | 6 | 15 | 2.58 | 0.99 | 485 | 80.83 | 31.66 |
| **Infant 22** | 13 | 45 | 3.44 | 1.69 | 1418 | 109.08 | 53.55 |
| **Infant 23** | 10 | 21 | 2.1 | 0.6 | 665 | 66.5 | 18.55 |
| **Infant 24** | 7 | 38 | 5.43 | 3.32 | 1173 | 167.57 | 102.16 |
| **Infant 25** | 14 | 47 | 3.35 | 1.33 | 1340 | 95.71 | 52.52 |
| **Infant 26** | 16 | 39 | 2.44 | 1.24 | 1265 | 79.06 | 40.43 |
| **Infant 27** | 13 | 35 | 2.66 | 1.28 | 1086 | 83.54 | 39.68 |
| **Infant 28** | 13 | 34 | 2.64 | 1.29 | 1086 | 83.54 | 40.88 |
| **Infant 29** | 11 | 46 | 4.2 | 2.79 | 1445 | 131.36 | 86.18 |
| **Infant 30** | 11 | 31 | 2.81 | 1.19 | 947 | 86.09 | 33.13 |
| **Infant 31** | 11 | 28 | 2.55 | 1.11 | 878 | 79.82 | 36.4 |
| **Infant 32** | 22 | 57 | 2.6 | 0.9 | 1855 | 84.32 | 29.29 |
| **Infant 33** | 8 | 17 | 2.18 | 0.84 | 563 | 70.38 | 27.11 |
| **Infant 34** | 21 | 58 | 2.78 | 0.96 | 1864 | 88.76 | 30.87 |
| **Infant 35** | 21 | 54 | 2.57 | 1.25 | 1758 | 83.71 | 41.51 |
| **Infant 36** | 12 | 41 | 3.44 | 1.85 | 1294 | 107.83 | 57.81 |
| **Infant 37** | 4 | 14 | 3.52 | 2.37 | 451 | 112.75 | 76.32 |
| **Infant 38** | 13 | 38 | 2.89 | 1.58 | 1187 | 91.31 | 49.6 |
| **Infant 39** | 9 | 28 | 3.13 | 1.25 | 887 | 98.56 | 39.6 |
| **Infant 40** | 10 | 54 | 5.43 | 3.06 | 1684 | 168.4 | 96.51 |
| **Infant 41** | 10 | 27 | 2.68 | 1.07 | 831 | 83.1 | 37.44 |
| **Infant 42** | 13 | 45 | 3.44 | 2.05 | 1431 | 110.08 | 65.69 |
| **Infant 43** | 9 | 24 | 2.65 | 1.56 | 755 | 83.89 | 49.62 |
| **Infant 44** | 11 | 42 | 3.86 | 1.89 | 1333 | 121.18 | 59.78 |
| **Infant 45** | 21 | 61 | 2.91 | 1.1 | 2009 | 95.67 | 36.99 |
| **Infant 46** | 11 | 43 | 3.93 | 2.44 | 1360 | 123.64 | 77.22 |
| **Infant 47** | 8 | 21 | 2.64 | 1.27 | 670 | 83.75 | 40.52 |
| **Infant 48** | 9 | 27 | 3.03 | 1.26 | 848 | 94.22 | 40.36 |
| **Infant 49** | 18 | 53 | 2.97 | 1.39 | 1704 | 94.67 | 45.38 |
| **Infant 50** | 13 | 40 | 3.1 | 1.33 | 1267 | 97.46 | 41.28 |
| **Infant 51** | 12 | 54 | 4.48 | 2.68 | 1670 | 139.17 | 85.2 |
| **Infant 52** | 9 | 26 | 2.84 | 1.47 | 812 | 90.22 | 48.22 |
| **Infant 53** | 9 | 25 | 2.77 | 1.5 | 785 | 87.22 | 48.97 |
| **Infant 54** | 9 | 26 | 2.89 | 0.97 | 831 | 92.33 | 31.56 |
| **Infant 55** | 14 | 38 | 2.71 | 1.45 | 1203 | 85.93 | 45.73 |
| **Infant 56** | 20 | 58 | 2.9 | 1.3 | 1860 | 93 | 41.98 |
| **Infant 57** | 13 | 37 | 2.87 | 1.65 | 1159 | 89.15 | 51.43 |
| **Infant 58** | 10 | 46 | 4.63 | 2.48 | 1442 | 144.2 | 75.96 |
| **Infant 59** | 9 | 30 | 3.38 | 1.89 | 974 | 108.22 | 60.52 |

**Table S3.** Minimum and maximum Elo-ratings within each group-year combination

| **Group** | **Year** | **min Elo-rating** | **max Elo-rating** |
| --- | --- | --- | --- |
| stu | 2011 | 542 | 1842 |
| mot | 2018 | 338 | 2201 |
| mst | 2018 | 374 | 2028 |
| sst | 2018 | 440 | 1883 |
| mot | 2022 | 393 | 2281 |
| ms1 | 2022 | 594 | 1487 |
| ms2 | 2022 | 875 | 2323 |
| ms3 | 2022 | 196 | 1050 |
| sst | 2022 | 304 | 1924 |

**Model formula**

$$\begin{aligned} \text{behavior}&\sim\frac{exp(c_{D}+c_{DS}\cdot\text{infant sex})}{1+exp(c_{D}+c_{DS}\cdot\text{infant sex})} \\ & -(\frac{exp(c_{D}+c_{DS}\cdot\text{infant sex})}{1+exp(c_{D}+c_{DS}\cdot\text{infant sex})}\cdot\frac{exp(c_{A}+c_{AS}\cdot\text{infant sex})}{1+exp(c_{A}+c_{AS}\cdot\text{infant sex})} \\ & \cdot exp\left( -exp(c_{B}+c_{BS}\cdot\text{infant sex})\cdot exp\left( -exp(c_{C}+c_{CS}\cdot\text{infant sex})\cdot\text{infant age} \right) \right)) \\ & \cdot\frac{exp(c_{ME}\cdot\text{maternal experience}+c_{FA}\cdot\text{fruit availability}+c_{MR}\cdot\text{maternal rank})}{1+exp(c_{ME}\cdot\text{maternal experience}+c_{FA}\cdot\text{fruit availability}+c_{MR}\cdot\text{maternal rank})} \end{aligned}$$

Coefficients in the model formula are represented using the notation $c_{X}$, where the subscript $X$ corresponds to the associated predictor variable. $a$, $b$, $c$ and $d$ correspond to the parameters of the Gompertz function, $c_{ME}$ denotes the coefficient for maternal experience, $c_{FA}$ the coefficient for fruit availability, and $c_{MR}$ the coefficient for maternal rank.

**Starting values and priors for each model**

For the parameters of the Gompertz function, we selected the priors based on visual inspection of the data and used their means as the starting values to provide stable initialization for the MCMC chains. In all models, parameter b had a mean of 2.3 and a standard deviation of 2, while parameter c had a mean of 0 and a standard deviation of 1. The means of parameters a and d varied between models, as shown in Table 3, but each had an standard deviation of 1 across all models.

**Table S4.** Means of priors of parameters *a* and *d* in all models

| **Model** | **mean prior** $\boldsymbol{d}$ | **mean prior** $\boldsymbol{a}$ |
| --- | --- | --- |
| Proportion of proximity time | 4.6 | 0.85 |
| Proportion of contact time | 4.6 | 1.4 |
| Mother responsibility of maintaining proximity | 0.98 | 0.08 |
| Mother responsibility of maintaining body contact | 2.2 | 0.7 |
| Proportion of mother proximity initiation | 0.6 | 0.85 |
| Proportion of infant proximity initiation | -0.40 | -0.75 |
| Proportion of mother body contact initiation | 2.1 | 0.8 |
| Proportion of infant body contact initiation | -0.84 | 1.3 |
| Proportion of nursing time | 0.98 | 0.08 |
| Proportion of independent feeding time | -5 | -0.8 |
| Proportion of carrying time | -1.38 | -4.5 |
| Proportion of independent locomotion time | -5 | -0.85 |

**
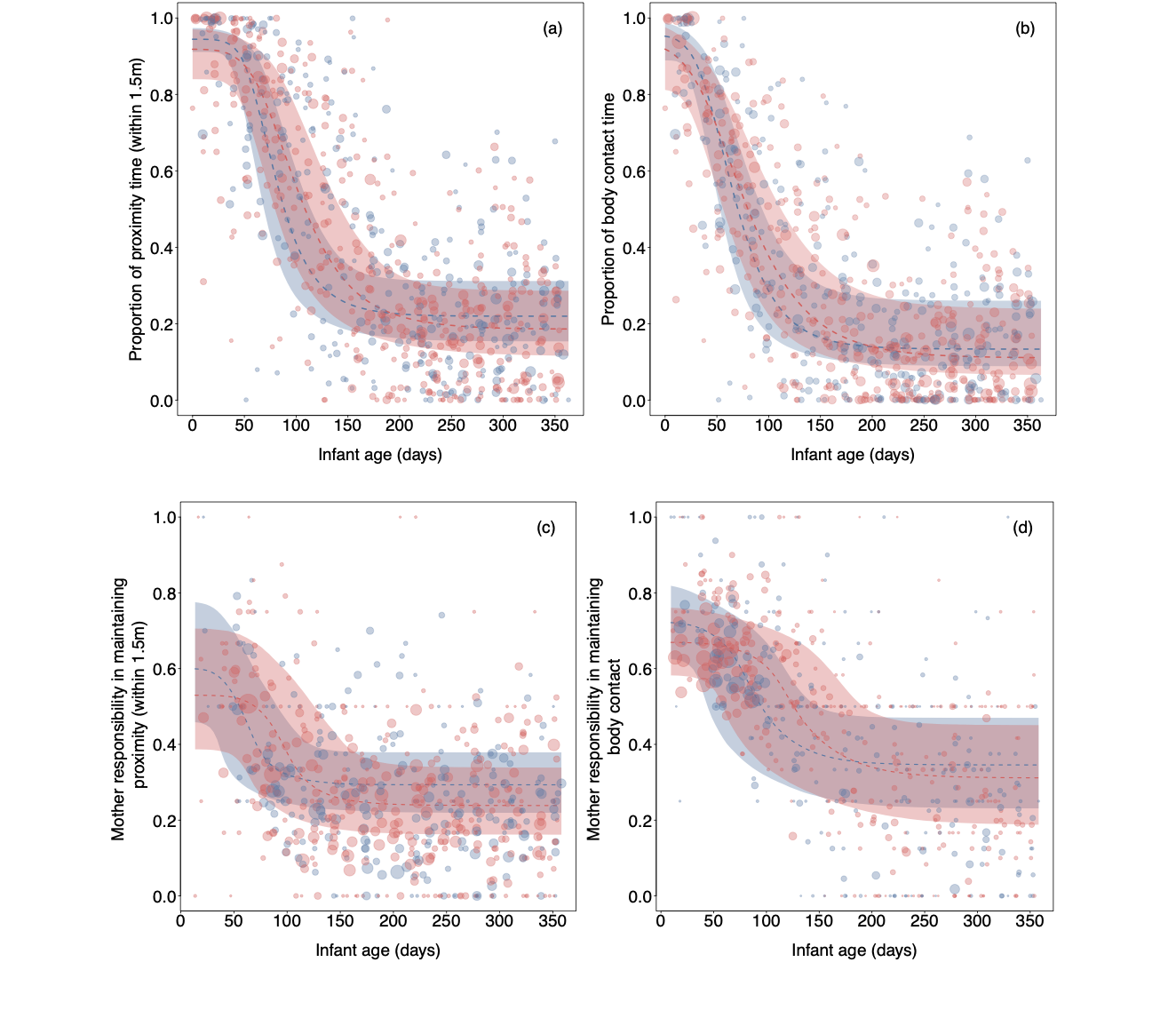
**

**Figure S2.** Interactive effects of infant age and sex on the proportion of time in mother-infant (a) proximity (within 1.5m) and (b) body contact, and the mothers’ responsibility in maintaining (c) proximity (within 1.5m) and (d) body contact. Dots represent block/infant data points, with the area of each dot being proportional to the observation effort for that data point (proportion of proximity/contact time: *min*=1.06 h, *max*=12.37 h; mother’s responsibility in maintaining proximity/body contact: *min*=2, *max*=295). The dashed line represents the median (50th percentile) of the posterior samples at each age value, while the shaded region corresponds to the 95% credible interval of the model predictions, determined as the 2.5th and 97.5th percentiles of the posterior samples. Blue dots, lines, and shaded regions are depicting males, and females are depicted in red.


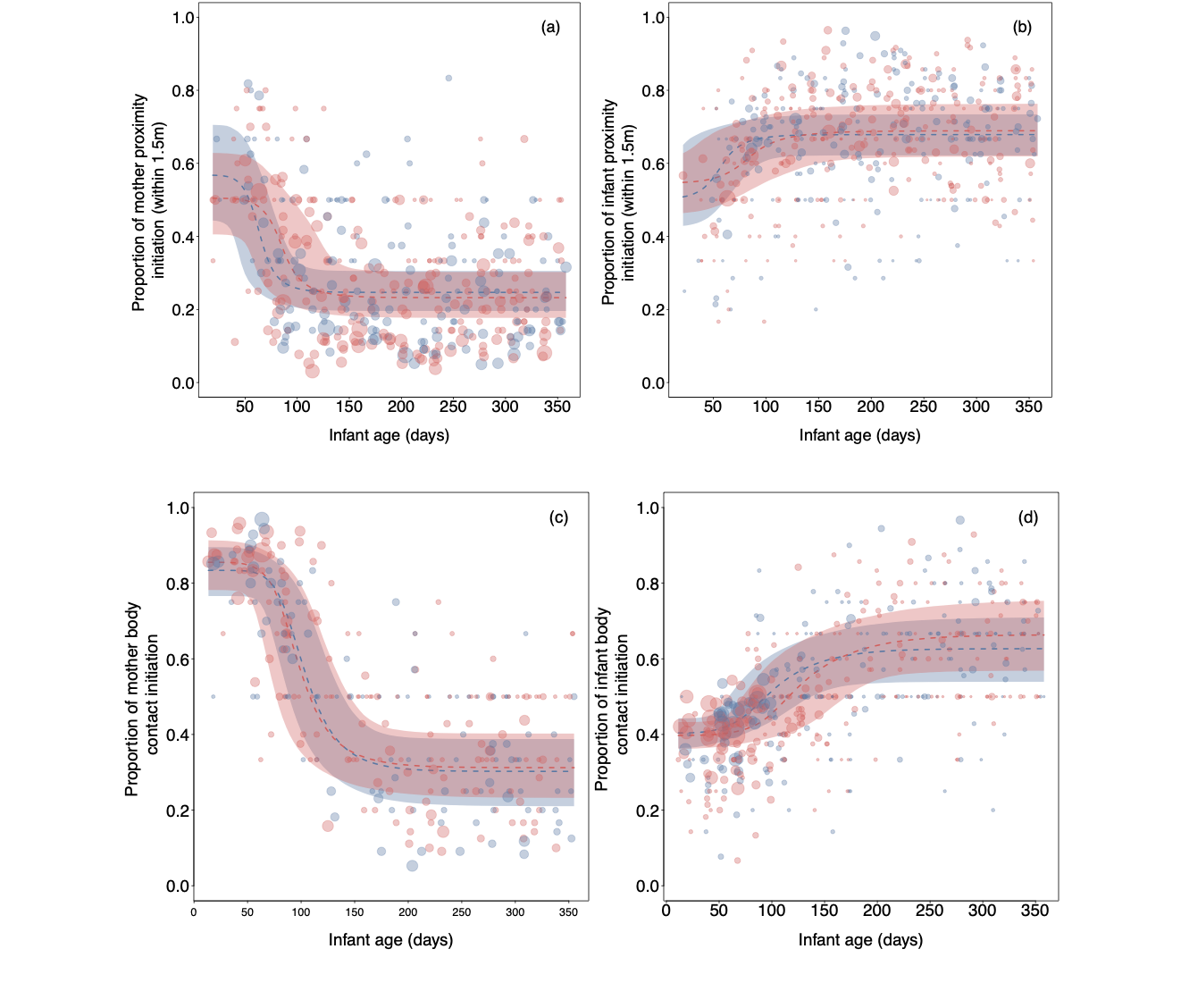


**Figure S3.** Interactive effects of infant age and sex on the proportion of (a) mother proximity initiation (within 1.5 m), (b) infant proximity initiation (within 1.5 m), (c) mother body contact initiation, and (d) infant body contact initiation. Proportions are calculated relative to all proximity or body contact changes initiated by the same member of the dyad. Dots represent period/infant data points, with the area of each dot proportional to the observation effort for that data point (*min*=2, *max*=236). The dashed line represents the median (50th percentile) of the posterior samples at each age value, while the shaded region corresponds to the 95% credible interval of the model predictions, determined as the 2.5th and 97.5th percentiles of the posterior samples. Blue dots, lines, and shaded regions are depicting males, and females are depicted in red.


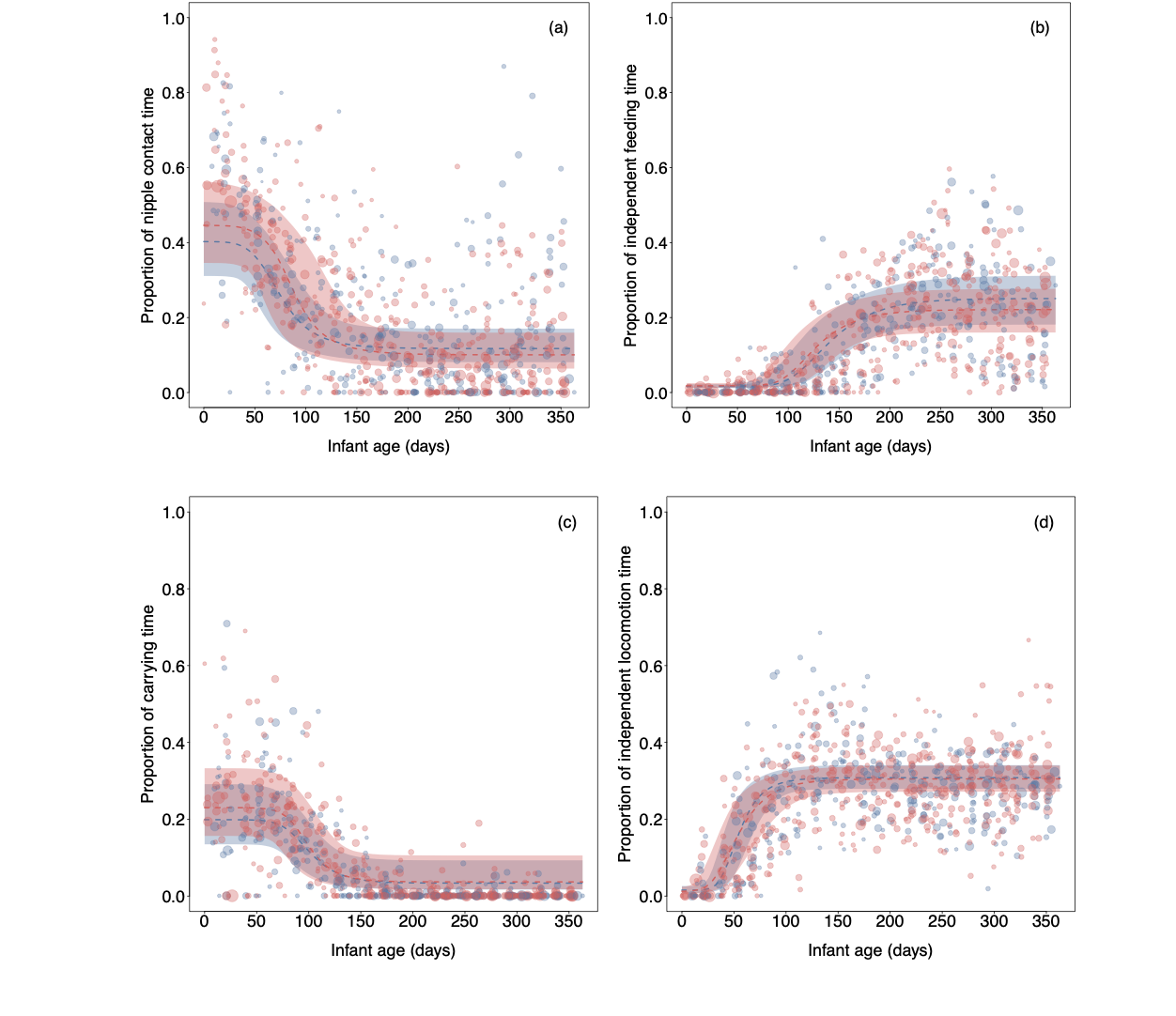


**Figure S4.** Interactive effects of infant age and sex on the proportion of time in (a) nipple contact, (b) carrying, (c) independent feeding, and (d) independent locomotion. Dots represent period/infant data points, with the area of each dot proportional to the observation effort for that data point (*min*=10 sampling points, *max*=389 sampling points). The dashed line represents the median (50th percentile) of the posterior samples at each age value, while the shaded region corresponds to the 95% credible interval of the model predictions, determined as the 2.5th and 97.5th percentiles of the posterior samples. Blue dots, lines, and shaded regions are depicting males, and females are depicted in red.

**
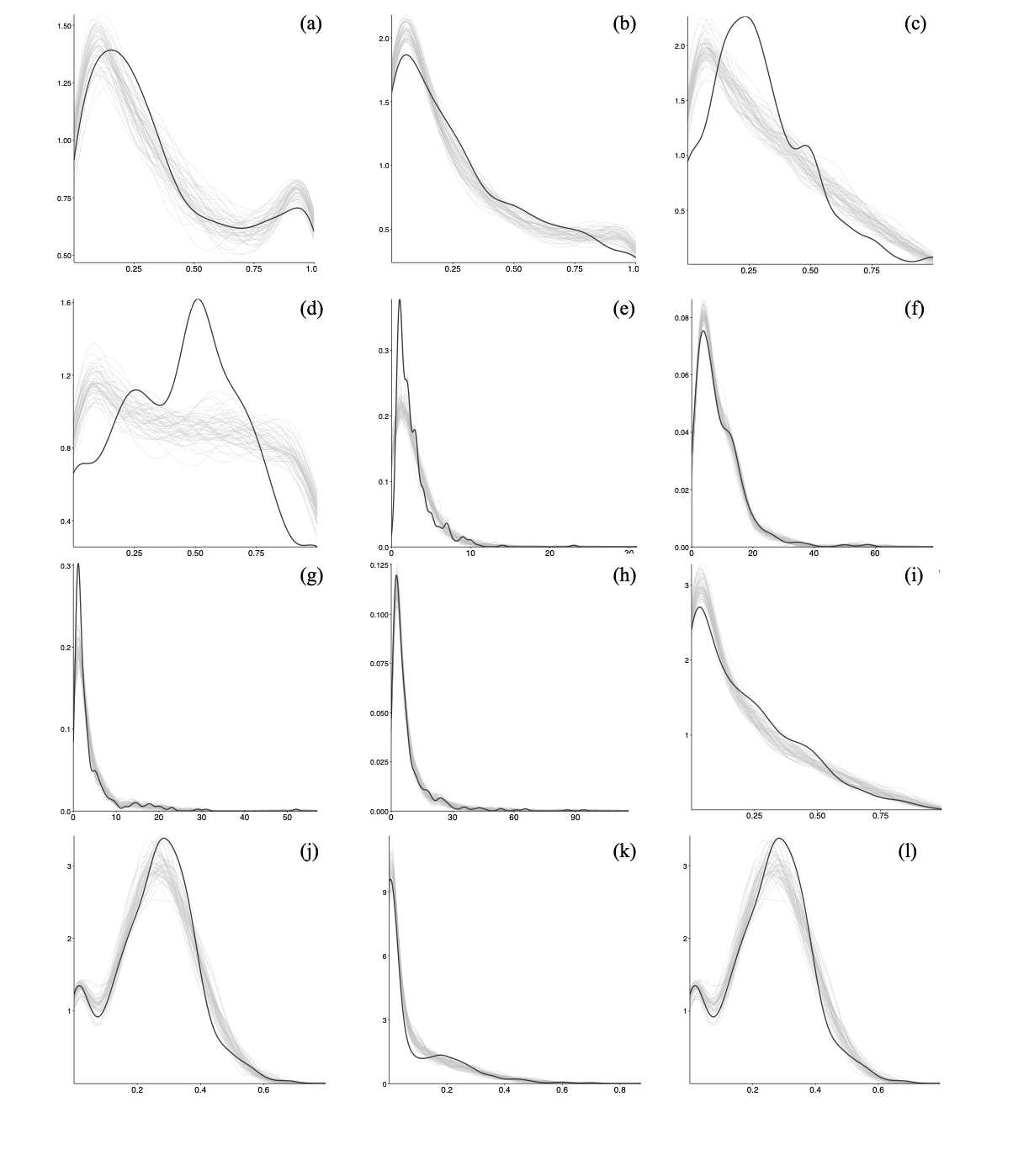
Figure S5.** Posterior predictive checks performed using the ppc_dens_overlay function from the Bayesplot R package, comparing the observed data with 50 posterior draws for: proportion of (a) proximity time and (b) contact time; mother’s responsibility in maintaining (c) proximity, and (d) body contact; proportion of (e) mother proximity initiation, (f) infant proximity initiation, (g) mother body contact initiation, (h) infant body contact initiation, (i) nipple contact time, (j) independent feeding time, (k) carrying time, and (l) independent locomotion time.

**
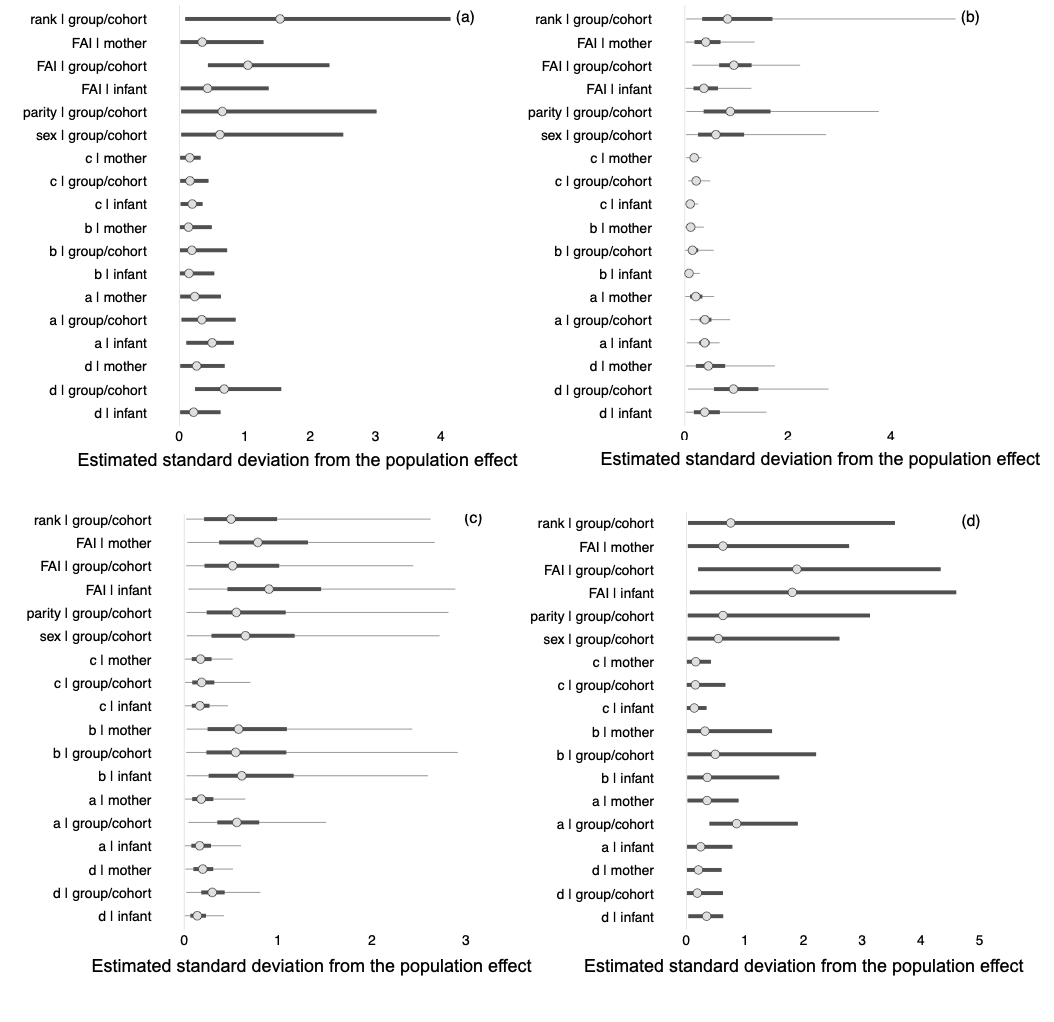
Figure S6.** Point estimates (50th percentile of the posterior probability distribution) for each random effect, with thin lines capturing 50% of the posterior probability and thick lines representing 95% posterior probability. Outcomes represent the proportion of time in mother–infant (a) proximity (within 1.5 m) and (b) body contact, and the mother’s responsibility in maintaining (c) proximity (within 1.5 m) and (d) body contact.


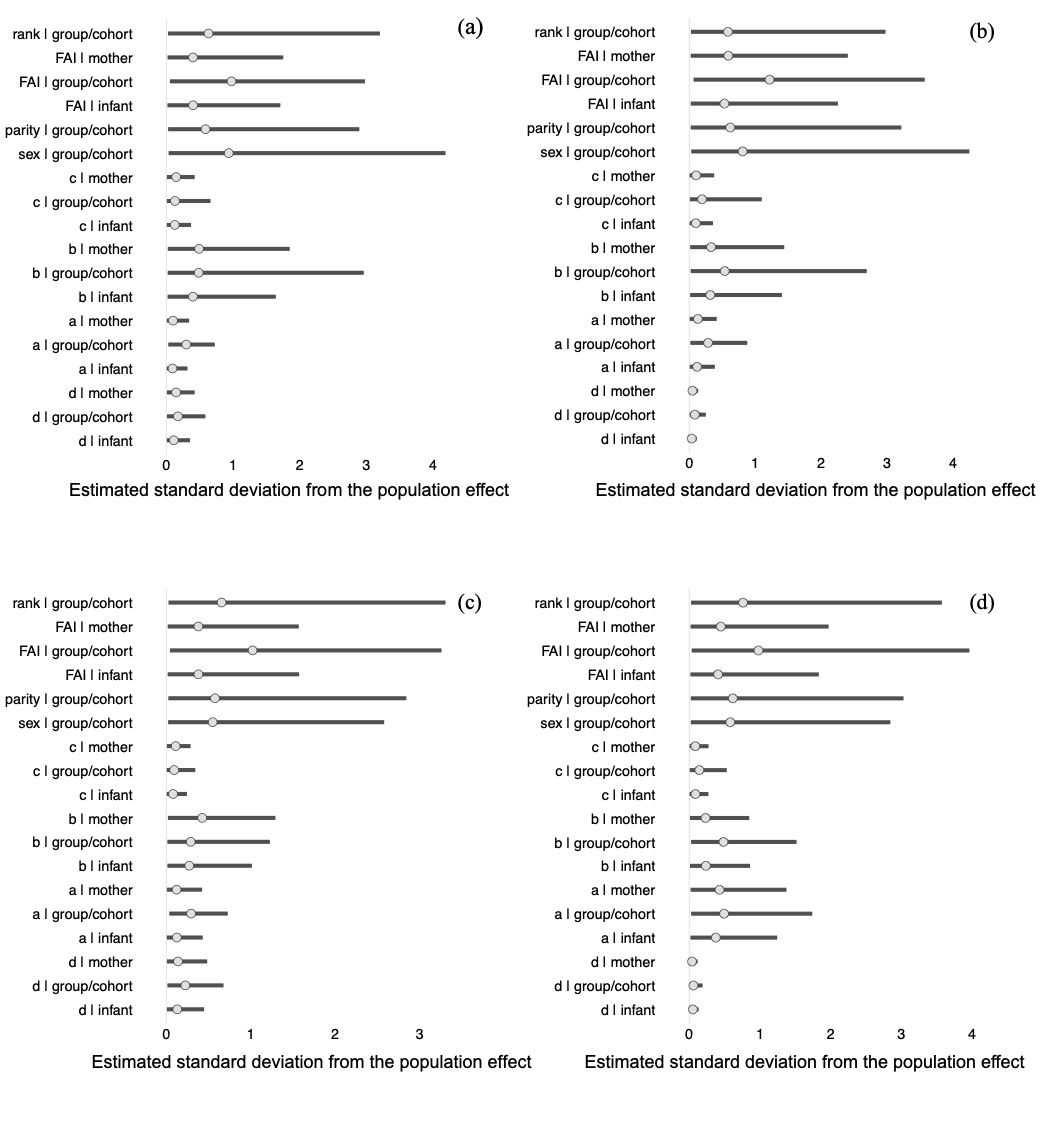


**Figure S7.** Point estimates (50th percentile of the posterior probability distribution) for each random effect, with thin lines capturing 50% of the posterior probability and thick lines representing 95% posterior probability. Outcomes represent the proportion of (a) mother proximity initiation (within 1.5 m), (b) infant proximity initiation (within 1.5 m), (c) mother body contact initiation, and (d) infant body contact initiation.

**
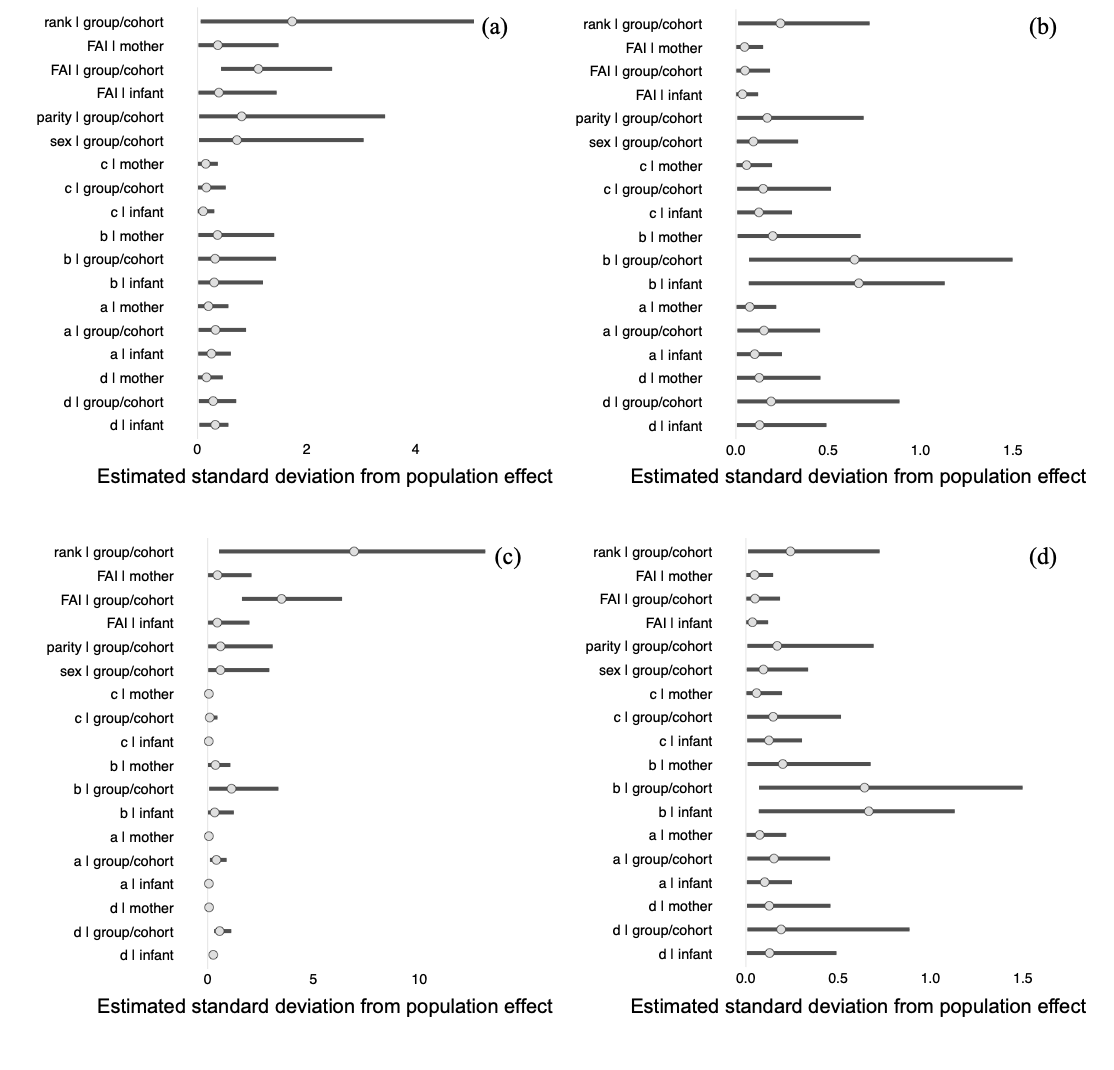
Figure S8.** Point estimates (50th percentile of the posterior probability distribution) for each random effect, with thin lines capturing 50% of the posterior probability and thick lines representing 95% posterior probability. Outcomes represent the proportion of time in (a) nipple contact, (b) carrying, (c) independent feeding, and (d) independent locomotion.

**Figure S9.** Posterior distribution of each infant deviation from the population-level effect in the proportion of proximity time (within 1.5 m) spent with the mother. Infants whose mother appears only once in the dataset are shown in grey. Infants who share a mother are depicted in the same color.

**Figure S10.** Dyadic age-trajectories for the proportion of proximity time (within 1.5 m) for each mother–infant dyad. Lines show the posterior median (50th percentile) of predicted proximity time across infant age for the focal dyad shown in each panel (purple), for all other dyads (grey) and for the population-level trajectory (black). The blue-shaded region indicates the 95% credible interval of the focal dyad’s posterior prediction. Dots represent period/infant data points, with the area of each dot proportional to the observation effort for that data point (*min*=1.06 h, *max*=12.37). Panels for infants whose mother appears only once in the dataset have grey borders, whereas panels for infants who share a mother use the same border color.

**Table S5.** Coefficients for the age-trajectory of the mother-infant spatial relationship (mean, standard deviations, and 95% credible intervals of posterior distribution)

| **Coefficient Estimate** | | **Standard**  **Error** | **95% CI**  **Lower** | **95% CI**  **Upper** |
| --- | --- | --- | --- | --- |
| **Proportion of proximity time** | |  |  |  |
| d | 2.79 | 0.40 | 2.14 | 3.73 |
| a | 1.76 | 0.23 | 1.34 | 2.26 |
| b | 2.31 | 0.33 | 1.73 | 3.03 |
| c | -3.59 | 0.16 | -3.89 | -3.26 |
| Sex (male) | 1.34 | 0.82 | 0.15 | 3.33 |
| Parity (nulliparous) | 2.15 | 1.17 | 0.06 | 4.72 |
| Fruit availability *^a^* | -1.31 | 0.56 | -2.50 | -0.29 |
| Maternal rank | 4.64 | 1.15 | 2.45 | 6.97 |
| **Proportion of contact time** | |  |  |  |
| d | 3.66 | 0.87 | 2.28 | 5.60 |
| a | 2.18 | 0.21 | 1.77 | 2.61 |
| b | 1.47 | 0.20 | 1.12 | 1.89 |
| c | -3.68 | 0.13 | -3.92 | -3.41 |
| Sex (male) | 3.13 | 1.14 | 1.26 | 5.66 |
| Parity (nulliparous) | 2.13 | 1.40 | -0.62 | 5.00 |
| Fruit availability *^a^* | -0.73 | 0.57 | -1.90 | 0.36 |
| Maternal rank | 7.09 | 1.23 | 4.63 | 9.49 |
| **Mother responsibility of maintaining proximity** | | | |  |
| d | 0.19 | 0.22 | -0.19 | 0.69 |
| a | 0.79 | 0.45 | 0.04 | 1.88 |
| b | 4.98 | 1.52 | 2.35 | 8.19 |
| c | -2.86 | 0.32 | -3.51 | -2.26 |
| Sex (male) | 1.60 | 0.98 | 0.00 | 3.86 |
| Parity (nulliparous) | 1.45 | 1.32 | -0.75 | 4.45 |
| Fruit availability *^a^* | -0.04 | 0.60 | -1.32 | 1.18 |
| Maternal rank | 1.68 | 1.03 | -0.13 | 3.93 |

**Table S6.** Coefficients for the age-trajectory of the transition from dependent to independent feeding and locomotion (mean, standard deviations, and 95% credible intervals of posterior distribution)

| **Coefficient Estimate** | | **Standard**  **Error** | **95% CI**  **Lower** | **95% CI**  **Upper** |
| --- | --- | --- | --- | --- |
| **Proportion of nipple contact time** | |  |  |  |
| d | -0.19 | 0.19 | -0.55 | 0.20 |
| a | 1.70 | 0.25 | 1.21 | 2.18 |
| b | 3.03 | 0.76 | 1.68 | 4.62 |
| c | -3.24 | 0.23 | -3.67 | -2.79 |
| Sex (male) | 1.83 | 1.10 | 0.18 | 4.55 |
| Parity (nulliparous) | 1.14 | 1.21 | -1.03 | 3.81 |
| Fruit availability *^a^* | -1.07 | 0.65 | -2.47 | 0.12 |
| Maternal rank | 4.29 | 1.45 | 1.62 | 7.27 |
| **Proportion of independent feeding time** | | |  |  |
| d | -2.72 | 0.14 | -3.30 | -2.45 |
| a | -0.92 | 0.10 | -1.11 | -0.73 |
| b | 5.04 | 0.85 | 3.59 | 6.92 |
| c | -3.22 | 0.17 | -3.54 | -2.87 |
| Sex | 0.44 | 0.58 | -0.51 | 1.76 |
| Parity (nulliparous) | 0.99 | 0.74 | -0.22 | 2.77 |
| Fruit availability *^a^* | -1.74 | 0.80 | -3.38 | -0.20 |
| Maternal rank | 1.91 | 1.14 | -0.22 | 4.28 |
| **Proportion of carrying time** | |  |  |  |
| d | -1.24 | 0.23 | -1.68 | -0.76 |
| a | 2.42 | 0.21 | 1.99 | 2.81 |
| b | 5.08 | 1.06 | 3.01 | 7.19 |
| c | -2.85 | 0.16 | -3.12 | -2.52 |
| Sex (male) | 3.92 | 1.15 | 1.85 | 6.35 |
| Parity (nulliparous) | 1.95 | 1.34 | -0.50 | 4.75 |
| Fruit availability *^a^* | -0.72 | 1.19 | -3.09 | 1.66 |
| Maternal rank | 5.06 | 2.16 | 0.76 | 9.20 |
